# Programmable manipulation of oligonucleotide–albumin interaction for elongated circulation time

**DOI:** 10.1093/nar/gkac156

**Published:** 2022-03-16

**Authors:** Cai Yang, Haitao Zhao, Yang Sun, Cheng Wang, Xinyao Geng, Ruowen Wang, Lumin Tang, Da Han, Jianjun Liu, Weihong Tan

**Affiliations:** Institute of Molecular Medicine (IMM), Department of Nuclear Medicine, Institute of Clinical Nuclear Medicine, State Key Laboratory of Oncogenes and Related Genes, Renji Hospital, School of Medicine, Shanghai Jiao Tong University, Shanghai 200127, China; Molecular Science and Biomedicine Laboratory (MBL), State Key Laboratory of Chemo/Biosensing and Chemometrics, College of Chemistry and Chemical Engineering, College of Biology, and Aptamer Engineering Center of Hunan Province, Hunan University, Changsha, Hunan 410082, China; Institute of Molecular Medicine (IMM), Department of Nuclear Medicine, Institute of Clinical Nuclear Medicine, State Key Laboratory of Oncogenes and Related Genes, Renji Hospital, School of Medicine, Shanghai Jiao Tong University, Shanghai 200127, China; Institute of Molecular Medicine (IMM), Department of Nuclear Medicine, Institute of Clinical Nuclear Medicine, State Key Laboratory of Oncogenes and Related Genes, Renji Hospital, School of Medicine, Shanghai Jiao Tong University, Shanghai 200127, China; Institute of Molecular Medicine (IMM), Department of Nuclear Medicine, Institute of Clinical Nuclear Medicine, State Key Laboratory of Oncogenes and Related Genes, Renji Hospital, School of Medicine, Shanghai Jiao Tong University, Shanghai 200127, China; Institute of Molecular Medicine (IMM), Department of Nuclear Medicine, Institute of Clinical Nuclear Medicine, State Key Laboratory of Oncogenes and Related Genes, Renji Hospital, School of Medicine, Shanghai Jiao Tong University, Shanghai 200127, China; Institute of Molecular Medicine (IMM), Department of Nuclear Medicine, Institute of Clinical Nuclear Medicine, State Key Laboratory of Oncogenes and Related Genes, Renji Hospital, School of Medicine, Shanghai Jiao Tong University, Shanghai 200127, China; Institute of Molecular Medicine (IMM), Department of Nuclear Medicine, Institute of Clinical Nuclear Medicine, State Key Laboratory of Oncogenes and Related Genes, Renji Hospital, School of Medicine, Shanghai Jiao Tong University, Shanghai 200127, China; Institute of Molecular Medicine (IMM), Department of Nuclear Medicine, Institute of Clinical Nuclear Medicine, State Key Laboratory of Oncogenes and Related Genes, Renji Hospital, School of Medicine, Shanghai Jiao Tong University, Shanghai 200127, China; Institute of Molecular Medicine (IMM), Department of Nuclear Medicine, Institute of Clinical Nuclear Medicine, State Key Laboratory of Oncogenes and Related Genes, Renji Hospital, School of Medicine, Shanghai Jiao Tong University, Shanghai 200127, China; Institute of Molecular Medicine (IMM), Department of Nuclear Medicine, Institute of Clinical Nuclear Medicine, State Key Laboratory of Oncogenes and Related Genes, Renji Hospital, School of Medicine, Shanghai Jiao Tong University, Shanghai 200127, China; Molecular Science and Biomedicine Laboratory (MBL), State Key Laboratory of Chemo/Biosensing and Chemometrics, College of Chemistry and Chemical Engineering, College of Biology, and Aptamer Engineering Center of Hunan Province, Hunan University, Changsha, Hunan 410082, China

## Abstract

Oligonucleotide (ON) therapeutics are emerging as a new generation of medicine with tremendous potential, but their clinical translation is hampered by inferior stability and short circulation time in the human body. Here, we report a general approach to manipulating the interaction between ONs and albumin by modulating hydrophobicity. A series of DNA aptamer derivatives were designed and prepared by programmable synthesis as an ON library with a gradient of hydrophobic base ‘F’. *In vitro* experiments revealed that the introduction of two F bases at both ends of ONs enhanced the biostability without sacrificing biological activities, while the binding affinity toward albumin was dramatically increased with *K*_d_ in the range of 100 nM to 1 μM. *In vivo* imaging confirmed the immediate formation of the aptamer–albumin complex after the injection, and the circulation time of the aptamer was dramatically elongated owing to the enhanced biostability and retarded renal excretion. The programmable incorporation of the F base provides a general approach to regulating albumin-binding affinity and enhancing the stability of aptamers *in vivo*, conferring aptamer therapeutics prolonged circulation time to meet clinical requirements.

## INTRODUCTION

Oligonucleotide therapeutics (ONTs), including siRNA, miRNA, antisense oligonucleotides (ASO), aptamer-based pharmaceuticals and nucleic acid vaccines, are emerging as a new generation of medicine next to small molecule drugs and antibody therapeutics (1–7). ONs are primarily manufactured by computer-controlled DNA synthesizer (8) automatically with excellent batch-to-batch consistency and reproducibility, and more than that, they can be readily modified with functionalities for optimization in biological activities (9–12). The potential of ONTs is tremendous, but their clinical translation is hampered by fast degradation and renal excretion, as unmodified ONs stay in the circulation system for <1 h (13). Therefore, the success of ONTs for therapeutics is largely dependent on the delivery system (13–18). Accordingly, many technologies have been developed to improve the *in vivo* stability of oligonucleotides and slow down the renal excretion speed (19–21). Viral and polymeric delivery systems protect ONTs from fast excretion and degradation *in vivo*, but the systems themselves may cause immune responses and be involved in tolerability and safety issues (22–24). It was recently observed that the nanoparticles and polymers (polyethylene glycol, PEG) may trigger severe allergic reactions using the approved mRNA vaccine against COVID-19 (25). Chemical modification is the simplest and most powerful strategy and has led to the success of several ONTs in clinical practice (26,27). Oligonucleotides modified with phosphorothioate backbone or 2′-functionalities have increased stability for their resistance to nuclease degradation (9,12,19,20). The conjugation with functionalities such as cholesterol and GalNAc at the terminals gives ONs as pure biomolecules with improved *in vivo* stability, cellular uptake and other properties (10,11,13,28).

Aptamers are single-stranded oligonucleotides with a certain sequence, which bind to target molecules specifically (29–31). They have been termed as ‘chemical antibodies’ for their similar functions as an antibody, some of which had been developed as therapeutic molecules by inhibiting the proteins concerned with diseases (32,33). On the other side, aptamers selected by Cell-SELEX (34) are potential ligands for targeted drug delivery comparable to antibodies, hence many efforts had been developed to the construction of aptamer–drug conjugates (ApDCs) (35). However, clinical applications of aptamers are puzzled by their short half-life *in vivo* (36). The replacement of natural ATCG bases with artificial bases has led to the discovery of aptamers with excellent *in vivo* stability (37–40), and the conjugation with polyethylene glycol (PEG) may elongate the half-life of aptamer therapeutics (6). However, long circulation time still presents one of the most challenging issues for aptamer therapeutics to be addressed.

Human serum albumin is the major protein in blood circulation making up to 50% of plasma proteins. There are several hydrophobic pockets inside the global protein so it transports essential substrates such as fatty acids, hormones to cells (41,42). It is critical to evaluate the interaction with albumin for the development of clinical molecules because it can either be a native delivery system or an *in vivo* trapper to exotic matters (43,44). For example, the formulation of paclitaxel with albumin improves the water solubility and circulation time of the hydrophobic drug and decreases its side effect (45–47). We and other teams had conjugated ONs with hydrophobic functionalities such as Evans blue or lipid compositions to improve the delivery and efficacy (48–50), while the hydrophobic interactions of such functionalities with albumin are too strong to be regulated for the clinical requirement.

It’s challenging to manipulate the hydrophobic interactions with albumin in physiological environment, especially when the guest molecules are hydrophilic ONs. Solid-phase synthesis technology has facilitated the preparation of artificial ONs modified with diverse functionalities. In our previous work on artificial DNAs (35,51–53), functional ‘elements’ can be readily incorporated into oligonucleotides as efficiently as A, T, C and G elements by DNA synthesizer from corresponding phosphoramidites. When a single hydrophobic element such as 3,5- bis(trifluoromethyl)benzoyl moiety (F base) has a weak binding affinity to multiple pockets of albumin, it is possible to enhance the binding affinity of ONs by multiplying the F base in it. Therefore the interaction of ONs with albumin could be manipulated by programmable modification and the circulation time of modified ONs would be dramatically elongated without sacrificing their biological abilities. Herein we reported our results on structural optimization of aptamers with hydrophobic F base and demonstrated how the interaction with albumin can be manipulated to obtain optimum aptamer with elongated retention time in blood.

## MATERIALS AND METHODS

### General Information

Unless otherwise noted, all reagents were purchased commercially without further treatment. General chemical reagents were purchased from Tansoole (Shanghai) Co., Ltd., J&K Scientific Ltd., and Sigma-Aldrich. For DNA synthesis, base and labeled reagents were purchased from Glen Research. 2-Cyanoethyl diisopropylchlorophosphoramidite, a general chemical for the introduction of phosphoramidite, was purchased from Energy Chemicals. General biological Kit and reagents were purchased from Beyotime Biotechnology. Ultrapure deionized water was used in all experiments, excluding organic synthesis, and was obtained from a Milli-Q Biocel system. Oligonucleotides were ordered from Sangon Biotech (Shanghai, China) and Suzhou Biosyntech. Modified DNA oligonucleotides were purified using reverse-phase HPLC (Agilent 1200) using a gradient of acetonitrile (5–80%) in 100 mM TEAA (pH 7.0). Concentrations of oligonucleotides were determined based on the absorbance at 260 nm. Concentrations of proteins were determined based on the absorbance at 280 nm. Gel images were captured and analyzed by GE Healthcare, Amersham Imager600 system. The binding affinity of ApDCs was studied by flow cytometry analysis (Beckman counter, Cytoflex), data analysis was performed using the FlowJo software (version X 10.0.7). Cell images were acquired with confocal microscopy (Leica SP8). Error bars in data plots were standard deviation (SD) with the experiments individually repeated in duplicates or triplicates. Synthesized small molecules were characterized by 1H and 13C NMR (500 MHz, Bruker).

### Molecular docking

To investigate potential binding sites of F base analog to albumin (PDB-ID: 1BM0) (54), automated molecular docking was performed using the ‘SwissDock program’. The results are scored and ranked by full fitness (kcal mol^−1^) and the spontaneous binding is exhibited by the estimated Gibbs free energy Δ*G* (kcal mol^−1^). Modeling results were visualized using UCSF Chimera v1.8 software.

### Cell culture

Human colon cancer cell line HCT116 was purchased from ATCC, Human leukemia cell Ramos was purchased from China Center for Type Culture Collection (CCTCC, China). The cells were cultured with ATCC-formulated RPMI-1640, supplemented with 10% fetal bovine serum, incubated at 37°C with 5% CO_2_ and 95% humidity.

### DNA sequences and buffers

Library sequence: 5′-NNNNNNNNNNNNNNNNNNNNNNNNNNNNNNNNNNNNNNNNN. Sgc8 sequence: 5′-ATCTAACTGCTGCGCCGCCGGGAAAATACTGTACGGTTAGA. Binding buffer was prepared with DPBS with 4.5 g/L glucose, 5 mM of MgCl_2_, 0.1 mg/ml of yeast tRNA and 1 mg/ml of BSA. Washing buffer was prepared with PBS with 4.5 g/L glucose and 5 mM of MgCl_2_.

### ^68^Ga-Labeling of NOTA-Sgc8 and NOTA-Sgc8-F23

For radiolabeling, ^68^GaCl_3_ solution (2 ml) was eluted from ^68^Ge/^68^Ga generator (IGG-100, Eckert & Ziegler AG) with 0.1 M HCl. Precursors NOTA-Sgc8 and NOTA-Sgc8-F23 were synthesized by conjugation of *p*-SCN-Bn-NOTA with amino-modified Sgc8 and Sgc8-F23 following established protocols. Then, precursors (10 nM) and sodium acetate aqueous solution (1 M, 300 μl) were added to ^68^GaCl_3_ solution. The reaction was incubated at 100°C for 20 min and purified with a NAP-25 column (GE) to obtain ^68^Ga-NOTA-Sgc8 and ^68^Ga-NOTA-Sgc8-F23.

### Flow cytometric analysis

To analyze the binding ability of aptamers, 250 nM of Sgc8 and Sgc8 derivatives labeled with fluorescein (FAM) were prepared and incubated with HCT116 cells in 200 μl of binding buffer at 4°C for 30 min. After incubation, non-specific aptamers were washed out, and then cells were resuspended in 200 μl washing buffer. A Backman flow cytometer was used to measure the fluorescence intensity of each sample.

### Stability analysis of aptamers in serum

About 250 nM FAM-labeled aptamers were incubated with RPMI 1640 with 10% fetal bovine serum (FBS) at 37°C. At designated time points, samples were heated at 95°C for 10 min to denature the enzyme and subsequently stored at −20°C until all samples were collected. Ten microliters DNA samples were mixed with 2 μl 6 × loading buffer (Sangon) and then loaded into 10% polyacrylamide gel in electrophoresis buffer (9 mM Tris, pH 8.0, containing 9 mM boric acid and 1 mM EDTA). After electrophoresis, the gels were analyzed with a molecular imager (GE Healthcare).

### The binding affinity of modified aptamers with human serum albumin

Human serum albumin was immobilized to 3.8 μm 40 mg/ml aldehyde/sulfate latex beads (Thermo Fisher Scientific, Waltham, MA USA) by mixing 1 ml of 5 mg/ml human serum albumin and 12 μl beads for overnight at 4°C with continuous vibration. The suspension was continuously vibrated for 30 min at room temperature. The reaction was stopped by adding 100 μl of 1 M glycine and 20% BSA/PBS and the mixture was kept under vibration for 1 h at room temperature. Afterward, a blocking solution containing 5% BSA and 1% yeast tRNA in DPBS was added and the solution was vibrated at room temperature for 1 h. The beads were washed in DPBS with 5 mM Mg^2+^ and centrifuged for 3 min at 6000 rpm twice. Washed beads were re-suspended in buffer and the suspension was divided into 21 tubes. Samples of Sgc8 and Sgc8-F23 labeled with FAM in gradient concentration were added to the suspension individually, and the mixtures were incubated at room temperature for 1 h. Finally, the beads were re-suspended in 200 μl DPBS with 5 mM Mg^2+^ after washing twice, which were used for binding affinity tests by flow cytometry.

### Confocal imaging

HCT116 cells were seeded in glass-bottom confocal dishes at a density of 1 × 10^5^ per well and incubated overnight. Cells were incubated with 500 nM of Sgc8 or Sgc8-F23 at 4°C for 30 min for binding ability. After binding, the aptamers were removed, then washed three times with washing buffer, and the medium with 10% FBS was added and incubated at 37°C for 1 h. The prepared samples were studied by confocal microscopy (Leica TCS SP8).

### Circular dichroism measurements

The CD spectra were measured using a Jasco-1500 CD spectrometer. Dissolved Sgc8 or Sgc8-F23 in DPBS containing Mg^2+^ (5 mM) to achieve a final concentration of 10 μM for 200 μl at room temperature. Accumulation of three scans from 320 to 200 nm using a 1 mm cell, a data pitch of 0.5 nm, a bandwidth of 2 nm and a scan speed of 50 nm/min.

### *In vivo* imaging and *ex vivo* biodistribution

Five-week-old female BALB/c nude mice were inoculated subcutaneously with 2 × 10^6^ HCT116 cells into the back of the right hind. When the volumes of HCT116 tumors reached 100–200 mm^3^, the tumor-bearing nude mice were randomly divided into groups and Cy5 labeled DNA (100 μl, 10 μM) were given respectively by the intravenous route via the tail vein. The fluorescence signal of Cy5 was obtained at different time points by an IVIS Lumina XR imaging system (Ex Filter: 620 nm; EM Filter: 670 nm). After imaging at 54 h after injection, mice were killed, tumors and major organs (hearts, lungs, spleens, livers and kidneys) were collected for imaging using an IVIS Lumina XR imaging system (Ex Filter: 620 nm; EM Filter: 670 nm). Then sliced tumors for fluorescence imaging (Leica TCS SP8).

### PET/CT imaging and biodistribution studies

PET/CT scans were performed using a Micro-PET/CT system (IRIS PET/CT, Inviscan, Strasbourg, France). HCT116 tumor-bearing mice were intravenously injected with about 3.7 MBq (100 μCi) of ^68^Ga-NOTA-Sgc8 and ^68^Ga-NOTA-Sgc8-F23 respectively, and sequential static PET scans were acquired at 4 h post-injection. For biodistribution study, mice with subcutaneous HCT116 xenografts were respectively injected with 0.37 MBq (10 μCi) of ^68^Ga-NOTA-Sgc8 and ^68^Ga-NOTA-Sgc8-F23 (*n* = 4 per group). Mice were sacrificed and dissected at 1 and 3 h p.i., and tumors were collected and weighed. The radioactivity of them was measured by a γ-counter. The results were presented as a percentage of injected dose per gram of tissue (%ID/g).

### *In vitro* cytotoxicity assay

*In vitro* cytotoxicity was determined using the Cell Counting Kit-8 (CCK8, Beyotime) assay with 96-well plates. HCT116 cells were seeded in 96-well plates with ∼10 000 cells in each well and incubated overnight for adherence. Cells were treated with free drug or ApDC in medium (without FBS). After 4 h, the medium was removed and a fresh medium with 10% FBS was added. The cells were then cultured for 72 h, and the CCK-8 assay was used to determine cell viability according to the standard protocol outlined by the manufacturer.

## RESULTS

### Design and synthesis of aptamer-derivatives modified with hydrophobic base

**Figure 1.**
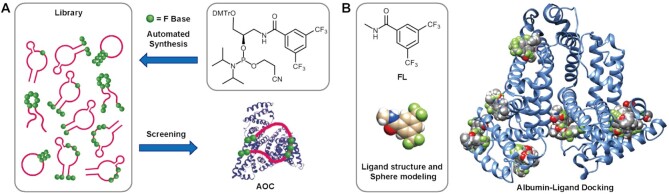
Rational design, synthesis, and screening of aptamer derivatives. (**A**) Synthesis of oligonucleotide library modified with F bases from phosphoramidite F and screening of an optimum molecule binding to albumin. (**B**) Molecular docking of F base analog with albumin.

As demonstrated by crystal structures of serum albumin complexes with small molecules, there are several binding sites for hydrophobic small molecular drugs, such as etoposide, idarubicin and bicalucamide (55–57). We had developed a phosphoramidite containing 3,5-bis(trifluoromethyl)benzene (F base) (Figure 1A), from which hydrophobic F can be incorporated into ONs as efficiently and conveniently as natural A, T, C and G bases in a programmable way (51). Hence we used molecular docking to predict the binding mode between albumin (54) and F base ligand using N-methyl-3,5-bis(trifluoromethyl)benzamide (FL, Figure 1B) as an analog. According to our initial docking result performed by SwissDock (58,59), there are multiple pockets of albumin that can be occupied by ligand FL with varied affinity (Figure 1B). Single-stranded aptamer-derivatives may bind to albumin with multivalent mode if they are modified with several hydrophobic F bases. Therefore, the interaction between ONs and albumin may be manipulated by modulating the number and sequence of F base, leading to the formation of the stable albumin–oligonucleotide complex (AOC, Figure 1A) in circulation and protecting ONs from fast excretion and degradation under physiological environment.

To verify the hypothesis, a series of aptamer-derivatives were designed and synthesized with different F base modifications, from which optimum derivatives can be screened out for clinical applications. It has been verified that the introduction of functionalities at 3′- or 5′-end of ONs would have the least effect on their biological activities. Besides, we had found that some part of aptamer Sgc8 sequence is compatible with the edition with other bases or functionality (60). Therefore, we designed a series of aptamer-derivatives introduced with F base (see Supporting Information for detailed sequence information, Supplementary Table S1) at the terminals and editable moiety of the aptamer, which were prepared as an ON library by a programmable DNA synthesizer using phosphoramidite F. The phosphoramidite was efficiently synthesized from commercially available 3-amino-1,2-propanediol and 3,5-bis(trifluoromethyl)benzoyl chloride in three steps, and the synthesis was readily scaled up to provide grams of the product (see SI for details, the structure of which was confirmed by ^1^NMR and ^31^P NMR spectra as Supplementary Figures S11 and S12). Phosphoramidite F was used to provide all the single-stranded DNAs (confirmed by mass spectra, Supplementary Figures S13–S33) smoothly with diverse F-modification. There was no obvious yield-decline even in the case of Sgc8-F07 or Sgc8-F27, into which there were 7 successive F bases incorporated.

### Screening of optimum aptamer by specificity and stability experiments

Sgc8 is the aptamer selected by cell-SELEX using CCRF-CEM cells as target cells and Ramos cells as negative cells (34), the target of which has been identified as membrane protein PTK7 (61). Overexpression of PTK7 has also been found in several other tumors such as HCT116 (62), so Sgc8 also binds strongly to HCT116 cells specifically. PTK7 protein has been an interesting target for antibody-drug conjugate (ADC) (63) and aptamer–drug conjugate development (64).

**Figure 2.**
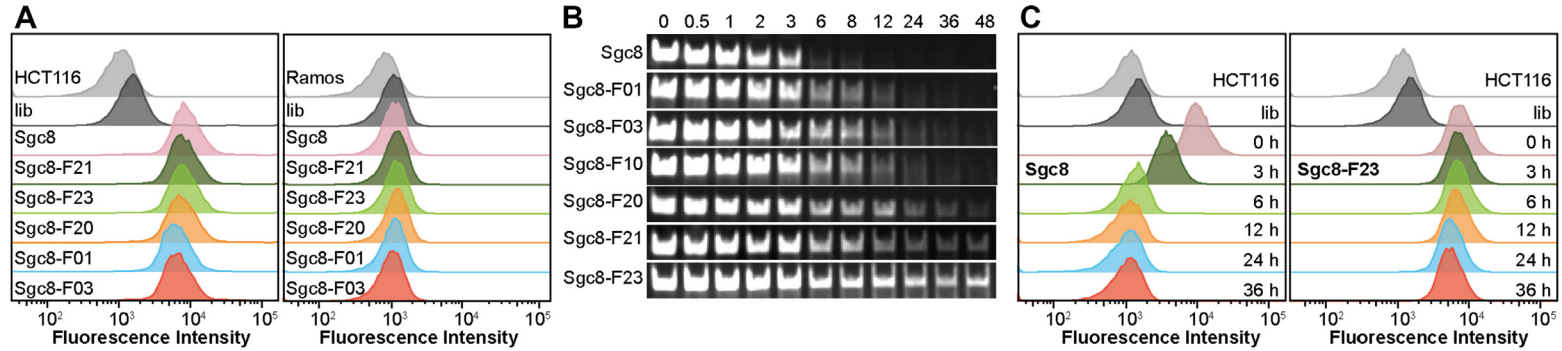
*In vitro* test on the specificity and stability of Sgc8. (**A**) Binding ability to HCT116 cells and Ramos cells tested with flow cytometric assay. Unmodified Sgc8 was used as the positive control, and library (lib) was used as the negative control. The cells were incubated with DNAs respectively at a concentration of 250 nM in a binding buffer for 30 min. The incubation temperature was 4°C. (**B**) Stability of Sgc8 variant after incubation with 10% FBS for 48 h analysis by PAGE. (**C**) Flow cytometric assay of HCT116 cells treated with Sgc8 and Sgc8-F23 after incubation with 10% FBS for 0–36 h, illustrating the binding ability after the stability test.

To investigate whether the incorporation of F base affects the binding specificity of aptamer, flow cytometry assay was performed using HCT116 cells as target and Ramos cells as negative control. Comparing the fluorescence intensity of both HCT116 and Ramos Cells with Sgc8, Sgc8-F10, Sgc8-F20, Sgc8-F30 and Sgc8-F40, the result suggests that the introduction of 1 to 4 F bases at both ends of Sgc8 have a negligible effect on the specificity of the aptamer (Figure 2A and Supplementary Figure S1A). Sgc8-F01, Sgc8-F03, Sgc8-F05 and Sgc8-F07 are the derivatives of Sgc8 in which natural bases are replaced with 1, 3, 5 and 7 F bases in the intermediate moiety correspondingly. The fluorescence experiments showed that they all bind to HCT116 comparable to Sgc8, but Sgc8-F07 has strong binding to Ramos cells. The introduction of 7 continuous F bases dramatically changed the hydrophobicity of the molecule and resulted in the nonspecific binding (Sgc8-F07 and Sgc8-F27, Supplementary Figure S1A). Simultaneous modification at the ends and intermediate moiety of Sgc8 may also maintain the specificity of the aptamer if the F base is incorporated properly. As shown by the experiments of Sgc8-F20, Sgc8-F21 and Sgc8-F23, they bind to HCT116 cells specifically against Ramos cell (Figure 2A and Supplementary Figure S2A).

Having demonstrated the specificity, then we tested the stability of the Sgc8 derivatives in the biological environment by incubation with 10% FBS. Modification of Sgc8 with one hydrophobic F base efficiently improved the stability of oligonucleotide, as shown by the gel results of Sgc8-F01 and Sgc8-F10 (Figure 2B). When Sgc8 was protected with two F bases at both 3′- and 5′- ends to give Sgc8-F20, Sgc8-F21 and Sgc8-F23, their lifetime in serum was dramatically increased up to 24 h or longer. However, when oligonucleotides were incorporated with 3, 4 or more F bases corresponding at both the 3′- and 5′- ends, their binding to proteins in FBS may be too strong to be dissociated during the PAGE process, as observed by the gel results of Sgc8-F30 and Sgc8-F40 (Supplementary Figure S1B). Strong binding to serum proteins was also observed for Sgc8-05, Sgc8-07 and Sgc8-27. PAGE gel experiments revealed that Sgc8-F23 is the most suitable oligonucleotide for *in vivo* imaging and targeted delivery of drugs, which is stable enough in a physiological environment. Sgc8-F20 may also be an optimum aptamer as a backup molecule of Sgc8-F23.

To further demonstrate the stability of Sgc8-F23, an integrated experiment was performed by detecting its specific binding ability with HCT116 cells after incubating with 10% FBS at different time intervals, and Sgc8 was used as a control. As shown in Figure 2C and Supplementary Figure S2B, specific binding of Sgc8 to the cells faded gradually and vanished finally after 6 h of incubation in FBS. In comparison, Sgc8-F23 kept strong binding to HCT116 cells even after 36 h of incubation, indicating its stability in biological environments. It was also tested by gel experiment that Sgc8-F23 is more stable than Sgc8 when they are incubated with nuclease (Supplementary Figure S1C).

### *In vitro* characterization of Sgc8-F23

**Figure 3.**
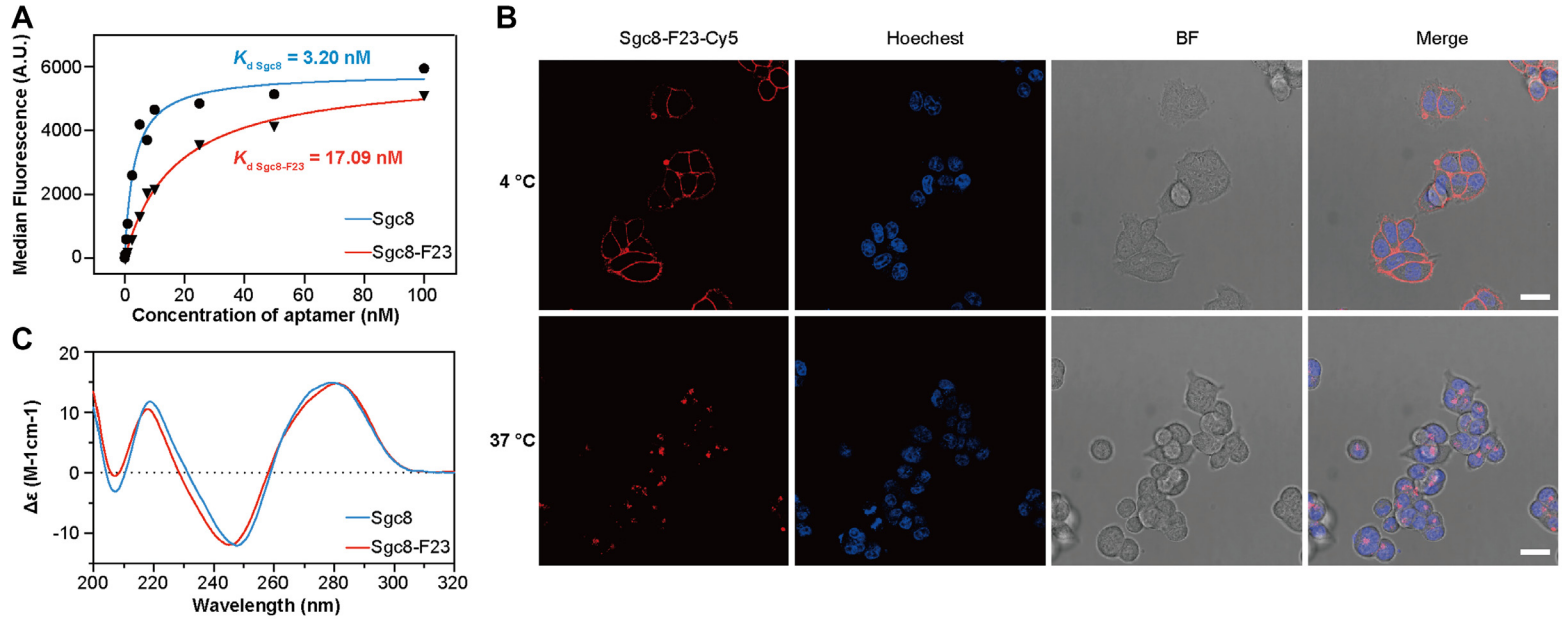
Characterization of Sgc8-F23. (**A**) The binding affinity to HCT116 of Sgc8-F23 and Sgc8 at 4°C. (**B**) Confocal microscopy fluorescence images of HCT116 cells treated with 500 nM Cy5-labeled Sgc8-F23 (red) in binding buffer at 4°C for 30 min or in culture medium (10% FBS) at 37°C for 1 h. The nuclei were counterstained with Hoechst 33342 (blue); scale bar, 20 μm. (**C**) Circular dichroism spectroscopy of Sgc8 and Sgc8-F23 in DPBS buffer with 5 mM MgCl_2_ at 25°C.

To demonstrate how the modification would change the properties of Sgc8, we subsequently performed a series of in vitro characterization of Sgc8-F23. First, binding data of Sgc8 and Sgc8-F23 with HCT116 cells were plotted together by Graphpad (Prism 8) in Figure 3A. The dissociation constant (*K*_d_) of Sgc8 and Sgc8-F23 with HCT116 cells was determined together by the equation:

$$\begin{equation*}{{Y}} = {{{B}}_{{\rm{max}}}}*X/\left( {{K_{\rm d}} + {{ X}}} \right)\end{equation*}$$

Where *Y* = the fluorescence from Cy5 of the ONs binding to a target protein, *X* = the concentration of the ONs, and *B*_max_ represents the maximum binding in the same units as *Y*. As shown in Figure 3A and Supplementary Figure S3, Sgc8-F23 maintained excellent binding affinity (*K*_d_ = 17.09 nM) to HCT116 cells, while the introduction of F base has a slightly negative effect on the binding affinity as compared to that of Sgc8 (*K*_d_ = 3.20 nM). The binding and internalization of Sgc8-F23 were characterized by confocal microscopy (Figure 3B). At 4°C, Sgc8-F23 mainly bound to the membrane proteins and located to the surface of HCT116 cells, which efficiently internalized into the cells at 37°C. This phenomenon was in line with previous research on Sgc8 and its ApDCs.

Circular dichroism (CD) is a sensitive tool to examine structural information, and 3D structure is critical for aptamers to recognize and bind to their targets. Therefore, CD spectroscopy of Sgc8-F23 was performed and compared with that of Sgc8 (Figure 3C). The spectra of Sgc8-F23 contained a negative Cotton band at 246 nm and a positive band with a maximum around 283 nm, which is consistent with those of Sgc8. The CD experiment demonstrated that Sgc8-F23 and Sgc8 formed similar geometric structures in an aqueous solution.

### The binding to serum albumin and *in vitro* competition with target cells

**Figure 4.**
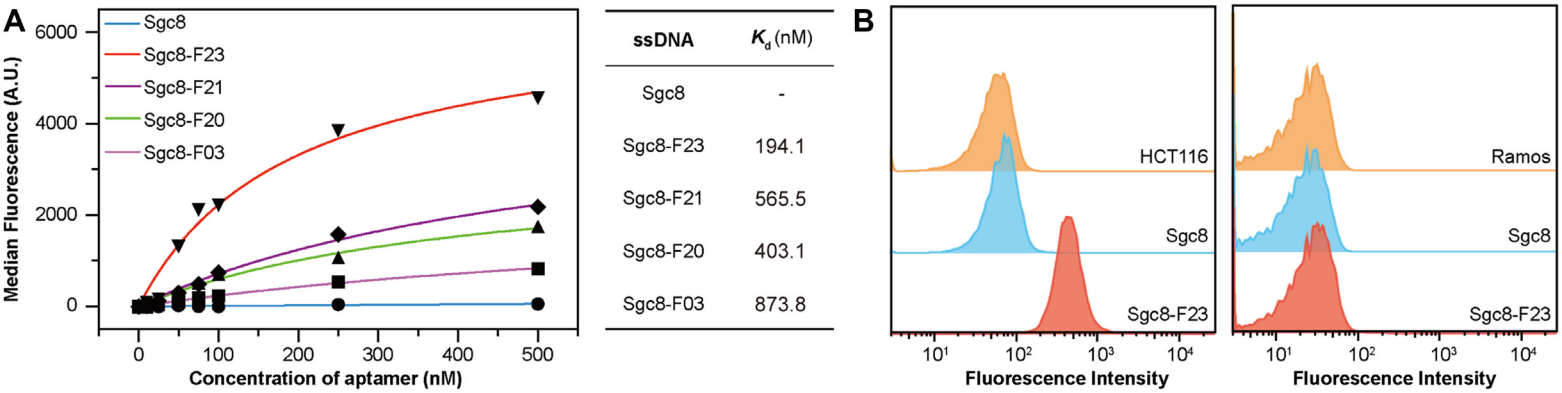
The binding affinity of Sgc8 derivatives to albumin. (**A**) Binding affinity analysis of Sgc8 derivatives and Sgc8 with aldehyde/sulfate latex beads coated HSA at room temperature. (**B**) HSA was immobilized to 3.8 μm 40 mg/ml aldehyde/sulfate latex beads (Thermo Fisher Scientific, Waltham, MA USA), after incubation with sgc8 or Sgc8-F23, the beads were washed to remove excess aptamers. The fluorescence signal of HCT116 and Ramos cells treated with the beads (sgc8 or Sgc8-F23 binding with HSA) was collected by flow cytometer.

According to the docking result, Sgc8 derivatives modified with F base may bind to albumin with varied affinity. Therefore, we tested the binding affinity of Sgc8, Sgc8-F23, Sgc8-F21, Sgc8-F20 and Sgc8-F03 with human serum albumin (HSA) by flow cytometry. The results revealed that unmodified Sgc8 rarely binds to HSA, but the derivatives bound tightly to the protein with dissociation constant (*K*_d_) values ranged from 100 to 900 nM. Sgc8-F23 exhibited the tightest-binding affinity to HSA, the *K*_d_ of which is 194.1 nM (Figure 4A and Supplementary Figure S4A–E). *In vitro* experiments demonstrated that the binding affinity of Sgc8-F23 to target HCT116 cells (*K*_d_ = 17.09 nM, Figure 3A) is 10-fold stronger than the affinity with HSA (*K*_d_ = 194.1 nM, Figure 4A). To demonstrate the competition between HSA and HCT116, Sgc8 or Sgc8-F23 were mixed with HSA respectively to form albumin–ON complex, and then the complex was incubated with HCT116 and Ramos cells respectively. Flow cytometry experiments were designed to illustrate the competition process. Fluorescence intensity was enhanced when HCT116 cells were incubated with albumin–Sgc8-F23 complex, but it wasn’t changed in the experiment of Ramos cells (Figure 4B and Supplementary Figure S4F). Therefore albumin–Sgc8-F23 complex formed in circulation may dissociate and release the aptamer molecule specifically when the complex reaches the proximity of HCT116 cells.

### *In vivo* experiments: specificity, circulation time and biodistribution

**Figure 5.**
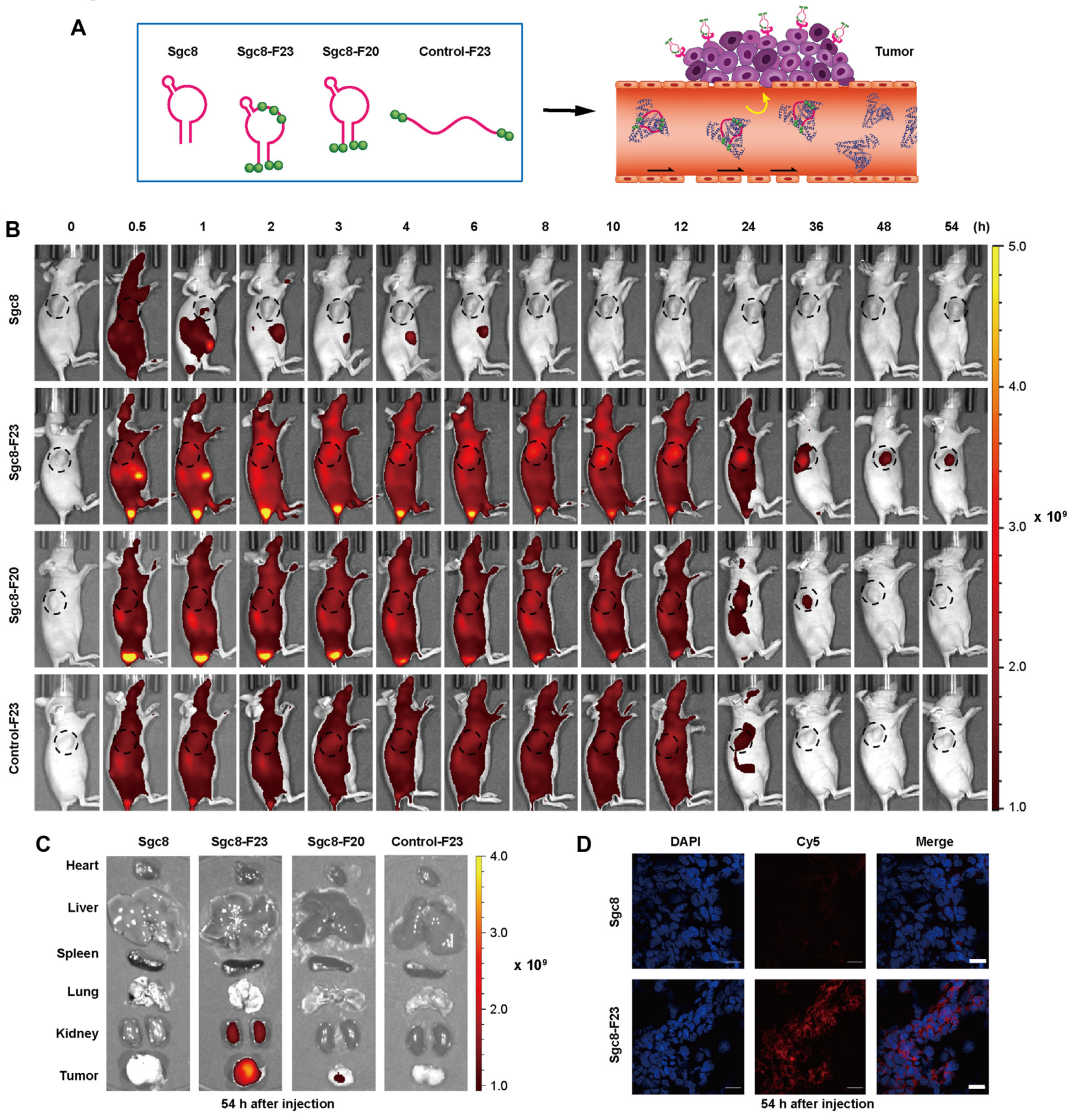
*In vivo* distribution of optimal modification of Sgc8. (**A**) Cy5-labeled aptamers were injected into mice to visualize the *in vivo* binding competition between albumin and tumor cells. (**B**) *In vivo* fluorescence imaging of HCT116 tumor-bearing mice after Cy5-labeled sgc8, Sgc8-F23, Sgc8-F20 or Control-F23 was injected through the tail vein; *n* = 3.(**C**) The distribution of the sgc8 and Sgc8-F23 in tumor and major organs (heart, liver, spleen, lung and kidney) at 54 h after injection visualized by fluorescence imaging; *n* = 3. (**D**) The representative fluorescent micrographs of the distribution of the sgc8 and Sgc8-F23 in the tumor at tissue level 54 h after injection were examined by confocal microscopy. Sgc8 and Sgc8-F23 were indicated by Cy5 (red). The nucleus was indicated by DAPI (blue). Scale bars, 20 μm.

As demonstrated by *in vitro* experiments, Sgc8-F23 and Sgc8-F20 bind to albumin forming a stable complex with *K*_d_ around 194.1 and 403.1 nM. Therefore, the optimum aptamers should form an albumin–oligonucleotide complex (AOC) immediately when they are injected into blood circulation. Besides, the binding affinity of Sgc8-F23 with target HCT116 cells was characterized to be remarkably stronger than that with albumin, so aptamers would be dissociated and target HCT116 cells when the complex is passing by (Figure 5A). AOC may protect aptamers from degradation and excretion in the circulatory system without sacrificing the targeting specificity. *In vivo* experiments were designed and performed to investigate the specificity and circulation time of the modified aptamers. Four groups of mice were injected with Cy5 dye-labeled Sgc8, Sgc8-F23, Sgc8-F20 and a control oligonucleotide Control-F23, respectively. The control oligonucleotide was a 45mer as Sgc8-F23, which was incorporated with the same amount of F bases at the position as Sgc8-F23. Consistent fluorescence imaging of the mice was performed from 0.5 to 54 h after the injection. Sgc8 was accumulated in the tumor, liver and kidney at 1 h, and was soon totally excreted out of the kidney, which was consistent with previously reported results. As a comparison, Sgc8-F23, Sgc8-F20, and the control were distributed throughout the whole body as long as 12 h from the imaging. It’s reasonable as these oligonucleotides modified with hydrophobic F bases can bind to albumin with good affinity, so the imaging represented the distribution of the albumin–oligonucleotide complex at the early period. It was also observed that Sgc8-F23 and Sgc8-F20 gradually accumulated in tumors, which might be a competing result between albumin and tumor cells. After 54 h, strong fluorescence was observed mainly in the tumor area of the mice injected with Sgc8-F23, while fluorescence signal from the mice group injected with Sgc8-F20 was observed as long as 36 h at the same scale (Figure 5B). The accumulation of Sgc8 and Sgc8-F23 in tumor at various time points post-injection was evaluated by measuring the fluorescent intensity of Cy5 (Supplementary Figure S5A), and the result clearly demonstrated the improved retention rate in tumor site with specificity for Sgc8-F23. Fifty-four hours post-injection, *ex vivo* imaging was performed to study biodistribution of the aptamer probes by Cy5 fluorescence detection. The results were consistent with that of *in vivo* imaging. At 54 h post-injection, the fluorescence signal of Sgc8-F23 was strong in tumor and kidney, and the signal of Sgc8-F20 was very weak in tumor and not found in other organs. In contrast, the fluorescence signals of Sgc8 and Control-F23 vanished completely in both the tumor and the organs (Figure 5C and Supplementary Figure S5B). Tumor tissue was observed by confocal microscopy, and a fluorescent micrograph indicated the accumulation of Sgc8-F23 in the tumor at 54 h after injection (Figure 5D). Results above indicated that the introduction of F bases prolonged the blood circulation of aptamer significantly.

### *In vivo* experiments by PET/CT imaging

**Figure 6.**
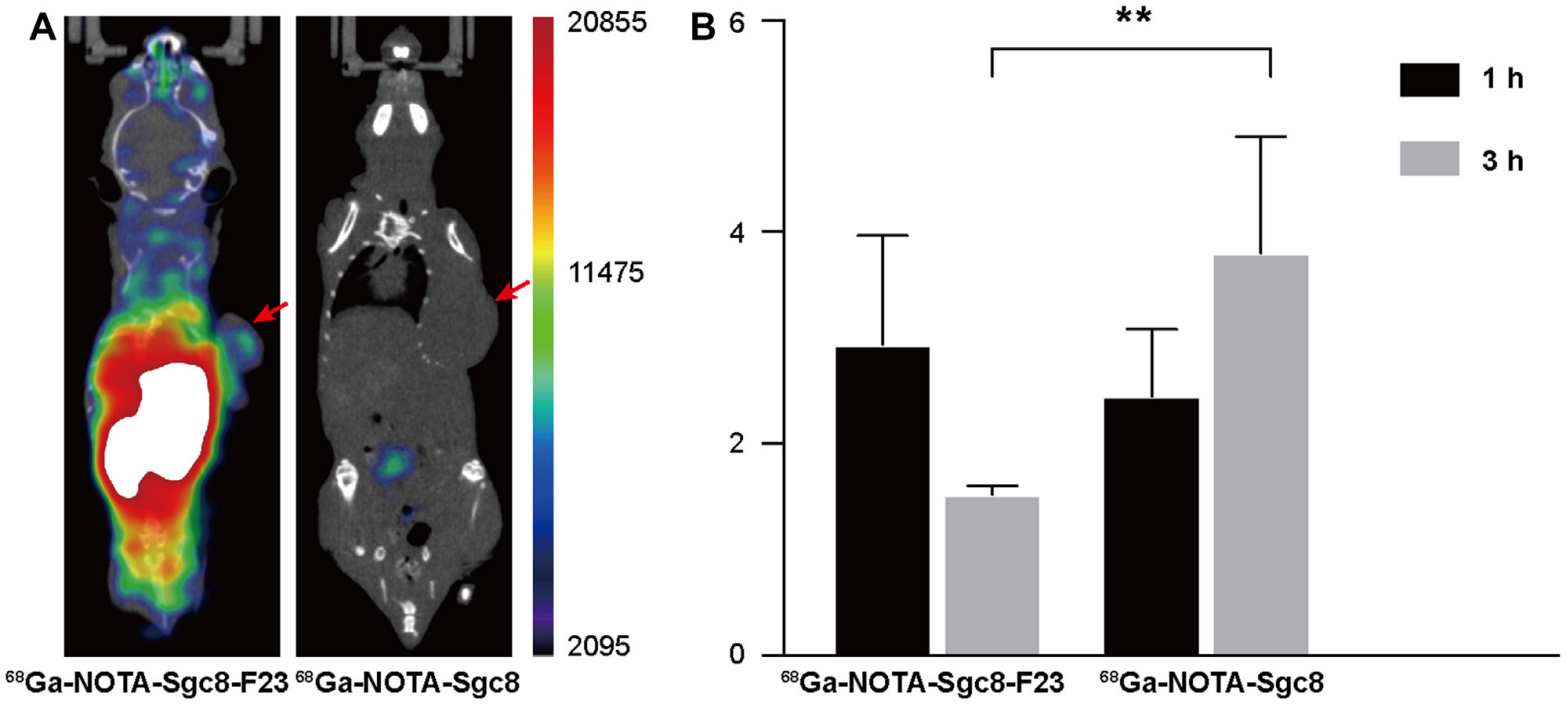
PET/CT imaging. (**A**) PET imaging of mice bearing subcutaneous HCT116 xenograft injected with ^68^Ga-NOTA-Sgc8 and ^68^Ga- NOTA-Sgc8-F23 at 4 h; Red arrow indicates the site of the tumor. (**B**) The distribution of the sgc8 and Sgc8-F23 in the tumor at 1 and 3 h after injection was measured by a γ-counter, and the results were presented as a percentage of injected dose per gram of tissue (%ID/g). *n* = 3, error bars denote standard deviation.

The *in vivo* behavior of Sgc8-F23 was further evaluated by PET/CT imaging and biodistribution study in the xenografted HCT116 mice model (Figure 6). NOTA-Sgc8-F23 was synthesized by established method for NOTA-Sgc8 (65). The radiolabeling of the precursor was monitored by RP-HPLC (the yields were given in Supplementary Figure S10), and the PET probes were purified for the imaging study. Mice were injected with ^68^Ga-NOTA-Sgc8 and ^68^Ga-NOTA-Sgc8-F23, respectively. Micro-PET/CT imaging of mice was performed at 4 h after the injection. Sgc8-F23 was still distributed throughout the body abundantly and it illuminated the tumor specifically, while unmodified Sgc8 was almost excreted out (Figure 6A). In the biodistribution study, the tumor uptake of ^68^Ga-NOTA-Sgc8-F23 increased gradually and was significantly higher than that of ^68^Ga-NOTA-Sgc8 at 3 h, while the uptake of ^68^Ga-NOTA-Sgc8 was decreasing over time (Figure 6B). Radiolabeling imaging is more sensitive and accurate than fluorescent labeling for *in vivo* imaging and biodistribution. The result showed in Figure 6B is coincident with that of Cy5-labeling Sgc8-F23, demonstrating the gradual accumulation and specificity of Sgc8-F23 to target tumor cells.

### The universality of F base modification

**Figure 7.**
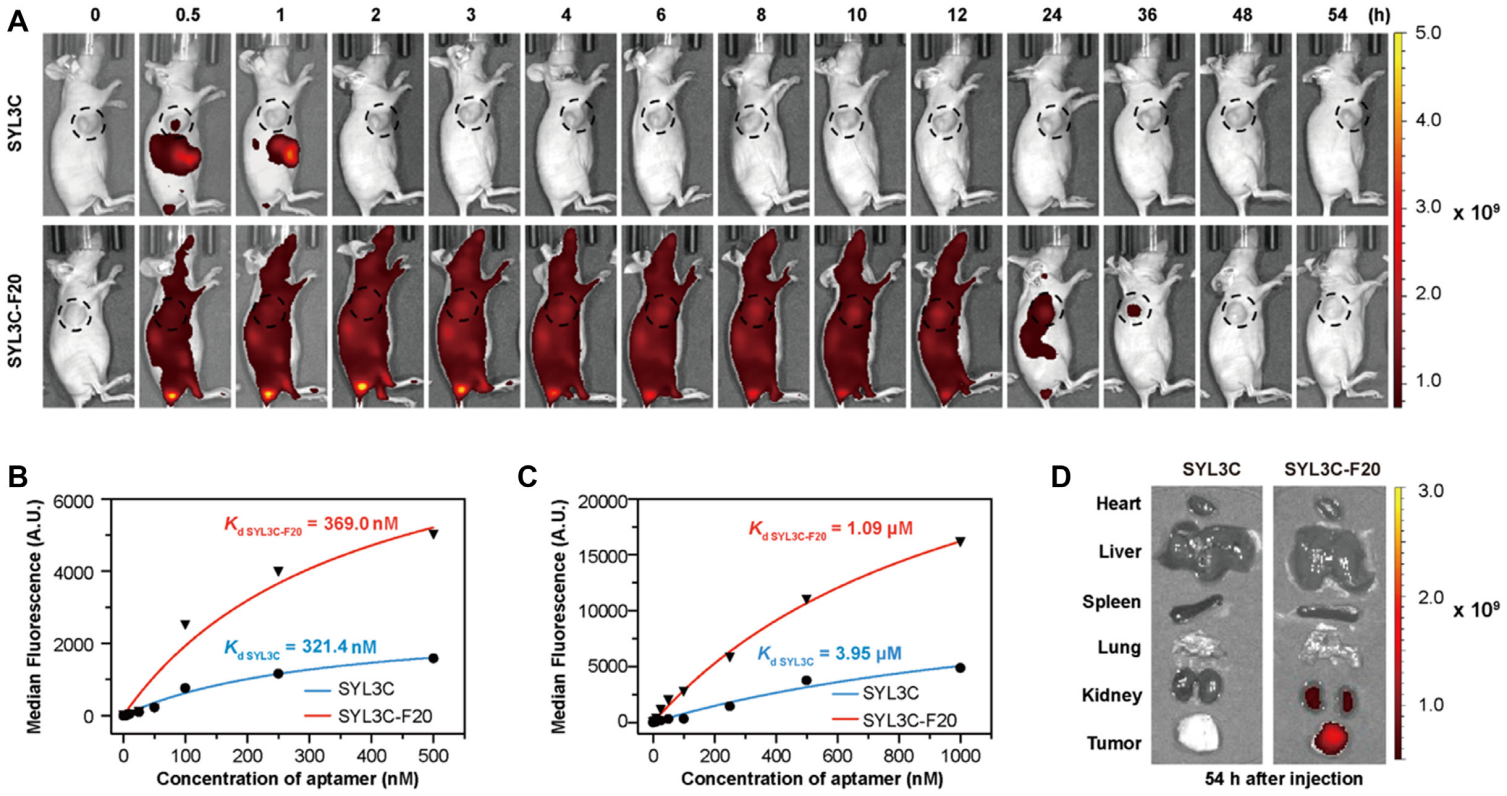
*In vivo* and *in vitro* studies of SYL3C and F-SYL3C. (**A**) *In vivo* fluorescence imaging of HCT116 tumor-bearing mice after Cy5-labeled SYL3C or F-SYL3C was injected through the tail vein. *n* = 3. (**B**) Binding affinity analysis of SYL3C and F-SYL3C with HCT116 cells at 4°C. (**C**) Binding affinity analysis of SYL3C and SYL3C-F20 with HSA at room temperature. (**D**) The distribution of the SYL3C and SYL3C-F20 in tumor and major organs (heart, liver, spleen, lung and kidney) at 54 h after injection was visualized by fluorescence imaging; *n* = 3.

Previous experiments have demonstrated that the incorporation of two F bases at both primes of oligonucleotides resulted in high binding affinity to albumin and increased biostability. This modification may be developed as a general method applicable to ONTs to enhance their circulating time and stability in the physiological environment. To testify to this hypothesis, we selected aptamer SYL3C (66) and modified it with two F bases at the primes to give SYL3C-F20, which was similarly characterized as Sgc8-F23 by *in vitro* and *in vivo* experiments (Figure 7; Supplementary Figures S6 and S7).

The target of aptamer SYL3C has been identified as EpCAM protein, which was also overexpressed as membrane protein on the surface of HCT116 (67,68). *In vivo* imaging experiments of HCT116 xenografts in mice were performed to illustrate the *in vivo* stability and circulation property. Cy5-labeled SYL3C and SYL3C-F20 were injected into two groups of mice respectively through the tail vein. From fluorescent signal SYL3C accumulated at the tumor site 0.5 h after the injection. The fluorescent signal could not be detected at the tumor site in 1 h, and it completely vanished in 2 h. Similar to Sgc8-F23, SYL3C-F20 was distributed all over the body at the beginning as it mainly stayed in the serum as an albumin complex. It began to accumulate at the tumor site from 1 h and gradually increased. Obvious signals were still detected in the tumor at 36 h, and it was completely diminished at 48 h (Figure 7A and Supplementary Figure S7A). The circulation time of SYL3C-F20 is shorter than that of Sgc8-F23, which may be resulted from their difference in binding affinity with albumin (*K*_d_ = 191.8 nm versus *K*_d_ = 1.09 μM). Analysis of the equilibrium dissociation constant (*K*_d_) by flow cytometry revealed that SYL3C-F20 still maintains binding affinity to HCT116 cells (*K*_d_ = 369.0 nM), which is close to the interaction of SYL3C (*K*_d_ = 321.4 nM) (Figure 7B and Supplementary Figure S7B). However, the binding affinity of SYL3C-F20 to albumin (*K*_d_ = 1.09 μM) is significantly stronger than that of SYL3C (Figure 7C and Supplementary Figure S7C). The tissue distribution was also evaluated by fluorescence imaging (Figure 7D and Supplementary Figure S7D). The fluorescence signal of SYL3C-F20 was observed in the tumor, and it was weaker in the kidney.

### Comparison of F base modification with cholesterol- and C18 lipid- modifications

**Figure 8.**
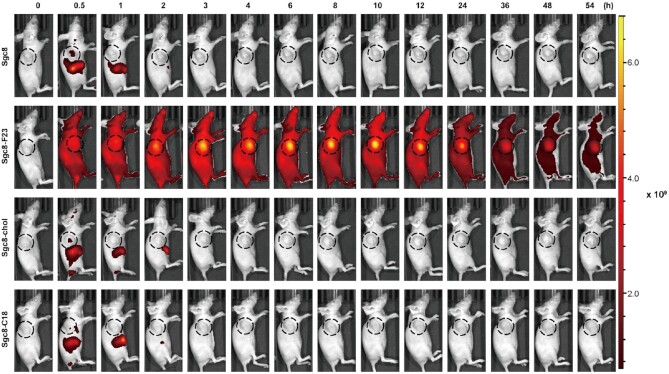
*In vivo* fluorescence imaging of HCT116 tumor-bearing mice after Cy5-labeled sgc8, Sgc8-F23, Sgc8-chol or Sgc8-C18 was injected through the tail vein; *n* = 3.

The introduction of lipids into ONs is a widely used technology that can facilitate cellular uptake and enhance the biostability of ONs. To understand the significance of programmable F base modification, aptamer Sgc8 analogs conjugated with cholesterol (Sgc8-chol) and linear C18 chain (Sgc8-C18) were prepared as controls. Flow cytometry assay demonstrated that the conjugation with cholesterol alters the binding affinity and specificity with HCT116 cells by comparing the fluorescence intensity of HCT116 cells with Sgc8 and Sgc8-chol (Supplementary Figure S8D). The result indicates that modification with cholesterol may not be suitable for aptamer-based targeted drugs. *In vivo* imaging experiments were performed to investigate the circulation property. Cy5-labeled Sgc8, Sgc8-F23, Sgc8-chol and Sgc8-C18 were injected into three groups of mice respectively through the tail vein. From fluorescent signal, Sgc8, Sgc8-chol and Sgc8-C18 accumulated at the tumor site at 0.5 h similarly after the injection, which diminished fast and couldn’t be detected in 2 h. In comparison, Sgc8-F23 was distributed all over the body at the beginning as it mainly stayed in the serum as an albumin complex for longer than 2 days (Figure 8 and Supplementary Figure S8A). The conjugation with lipids couldn’t elongate the circulation time of ONs from the *in vivo* imaging experiment. The tissue distribution was also evaluated by fluorescence imaging (Supplementary Figure S8B,C), and the result confirmed the observation.

### Cytotoxicity of aptamer–drug conjugates constructed with Sgc8-F23

**Figure 9.**
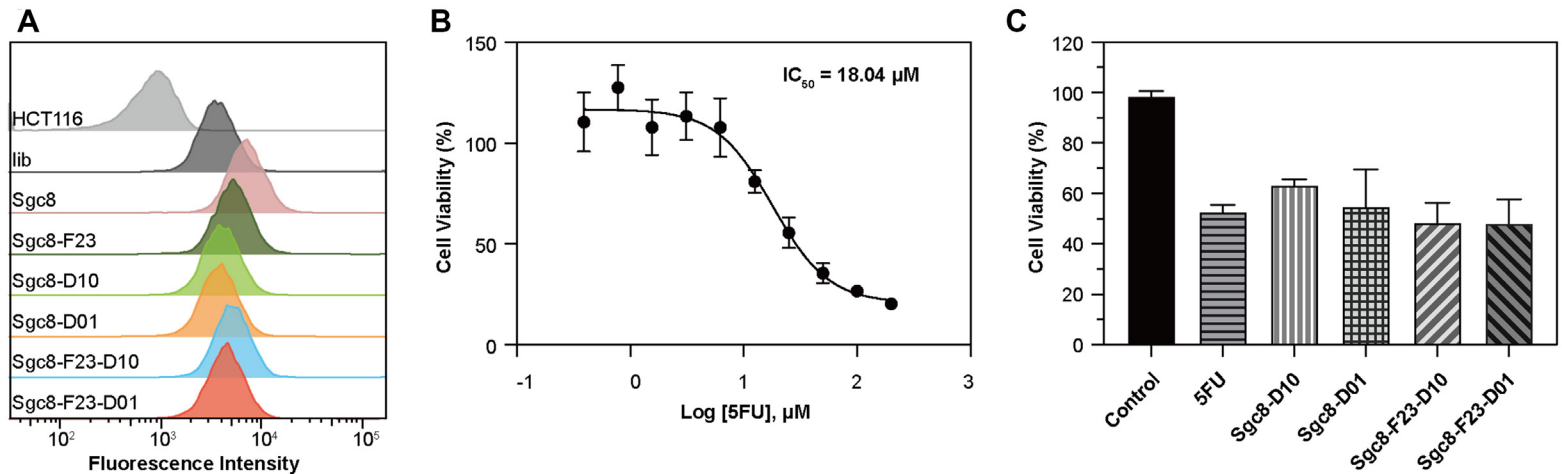
The cytotoxicity of optimal modification of Sgc8. (**A**) Flow cytometric results indicate the binding ability of ApDCs to target HCT116 cells. Lib was a negative control. All probes were labeled with FAM at the 3′ ends. The cells were incubated with DNAs respectively at a concentration of 250 nM in a binding buffer for 30 min. The incubation temperature was 4°C. (**B**) The IC_50_ value of 5FU against HCT116 cells was measured by CCK8 assay; *n* = 5. (**C**) The cytotoxicity of free 5FU and ApDCs on HCT116 cells was analyzed by CCK-8 assay. The concentration was 18.04 μM; *n* = 4. Error bars denote standard deviation.

Aptamer–drug conjugates are potential therapeutics in which drug molecules are delivered to the target specifically and followed by a controllable release *in situ*. The modification of aptamers with the F base may confer corresponding ApDCs enhanced pharmacokinetics and blood retention, and thus better efficacy. It is important to investigate whether the modification prevents drug release or not, which can be demonstrated by comparing the inhibitory activities of the ApDCs and drug molecules at the same concentration. Therefore, Sgc8-F23-D01 and Sgc8-F23-D10 were prepared in which 5-fluorouracil (5-FU) was conjugated at 5′-prime and the medium position of Sgc8-F23, respectively. Corresponding Sgc8-5FU conjugates were also prepared as a control for cytotoxicity study. First, the binding ability to HCT116 cells was examined through detecting the FAM fluorescence intensities by flow cytometry (Figure 9A and Supplementary Figure S9), and all of the ApDCs bound to target cells specifically. Then cytotoxicity of the ApDCs was studied together with 5-FU as a comparison. As the inhibitory concentration (IC_50_) of 5FU was tested to be 18.04 μM by CCK-8 assay (Figure 9B), HCT116 cells were incubated with 5FU and ApDCs for 72 hours respectively (Figure 9C) at the same concentration. Sgc8-F23-D10 and Sgc8-F23-D01 inhibited HCT116 cells with 50% cell viability, which is close to that of 5FU and Sgc8-D01. The cytotoxicity experiment results suggested that 5FU be released efficiently no matter how it was conjugated to Sgc8-F23.

## DISCUSSION

Drug-delivery technologies play important role in pharmaceuticals, as they enhance the delivery efficiency of therapeutics and minimize off-target side effects (69). However, the delivery system itself may cause nonspecific toxicity or serious allergy. Utilizing serum proteins as drug-delivery vehicles has been emerging as an advanced strategy with excellent biocompatibility, which has been demonstrated by FDA-approved Abraxane.

The clinical potential of ONTs is tremendous but they suffer from inferior stability and short retention time in blood circulation. Therefore, many drug-delivery technologies have been developed to improve the pharmacokinetic profiles for ONTs. The conjugation with hydrophobic functionalities generally enhances the *in vivo* stability of ONTs and this technology has been applied to the development of siRNA therapeutics. We and Sleiman group had identified that lipid-tailed ONs form an albumin–ON complex in circulation automatically by the hydrophobic interaction (49,70). However, the interaction between albumin and lipid ON is not strong enough to maintain the complex for drug-delivery purposes. In addition, the interaction is one-fold and couldn’t be modulated to meet different requirements for ONTs varied in molecular weight, sequences, secondary structure and target organs.

Although widely recognized as biological therapeutics, ONs are chemically synthesized molecules by DNA synthesizer, enabling them to be modified with functionalities precisely in a programmable manner. In the discovery process of small molecular drugs, identifying a lead compound is the start point, and tremendous structural optimization is performed as a key process to enhance the properties to meet the clinical requirement. When an aptamer has been selected and verified that binding to its antigen specifically as a ‘chemical antibody’, it may also be treated as a lead compound with potential in clinical applications. Similarly, structural optimization would be an important and necessary process to improve pharmacokinetic properties, which may be performed by programmable solid-phase synthesis using functional phosphoramidites. Yet there are quite limited functional phosphoramidites available, and principles are needed for structure-optimization of ONs to follow.

Herein we explored a functional phosphoramidite containing hydrophobic F base in structure–activity relationship study of aptamers. As predicted by SwissDock, the F base analog binds to albumin in several pockets with different affinities, therefore the interaction with albumin can be modulated by programmable incorporation of F bases at proper sites of the aptamer sequence (Figure 1). By solid-phase synthesizer, it’s convenient to generate a pool with a series of Sgc8 derivatives for screening. From flow cytometry and gel experiments, the specificity and stability of the derivatives were determined, and it was found that incorporation of two F bases at both primes of Sgc8 enhanced the stability significantly (Figure 2). More importantly, the binding affinity of ONs with albumin is tunable which is determined by the modality of F base modification. From the screening experiments, Sgc8-F23 and Sgc8-F20 were identified as the optimum derivatives and used for *in vivo* imaging. The study revealed that Sgc8-F23 and Sgc8-F20 were distributed throughout the whole mice body in the first 12 h, and they were gradually accumulated in the tumor meanwhile. It suggested these aptamers modified with F bases bind to albumin and form a stable complex compared with the imaging result of Sgc8, and they also displayed targeted releasing to tumor cells from the binding with albumin. After 54 h of injection, Sgc8-F23 was still accumulated in the tumor, which is longer than that of Sgc8-F20 (36 h). Sgc8-F23 is the derivative incorporated with three more F bases in the middle and exhibited better biostability and binding affinity to albumin than Sgc8-F20. From the *in vivo* experiment, we demonstrated that the interaction of aptamer with albumin can be manipulated by regulation of the F base, and the stability and retention properties in circulation were thus optimized correspondingly.

From the *in vivo* imaging of Sgc8-F23 and Sgc8-F20, we speculated that it might be a general approach to enhance stability and retention time in circulation for all aptamers by the introduction of two F bases at both primes of the oligonucleotides. Therefore, it is necessary to validate it with another aptamer. SYL3C was a widely used aptamer with identified antigens, which was selected and modified similarly to give SYL3C-F20 as the derivative. The retention time of SYL3C-F20 accumulated in the tumor was around 36 h, which is almost 40 times longer than that of SYL3C. The results demonstrated the generality of F-base modification for aptamer therapeutics.

In conclusion, we explored the approach to structural optimization of aptamers in a programmable manner. The incorporation of hydrophobic F bases at both primes conferred ONs excellent binding affinity to albumin and thus pharmacokinetic profiles of ONs could be optimized to meet the clinical requirement. In this way, we have developed optimum aptamers with a long retention time in the circulation system, which has translational potential as targeted drug-delivery vehicles and diagnostic PET probes.

## DATA AVAILABILITY

All the data supporting the findings of this study are available within the article and supplementary information file and from the corresponding author upon reasonable request. A reporting summary for this article is available as a Supplementary Information file. Source data are provided with this paper. The flow cytometry data from this publication have been deposited to FlowRepository database under IDs: FR-FCM-Z4RV, FR-FCM-Z4SZ, FR-FCM-Z4SY and FR-FCM-Z4S2.

## Supplementary Material

gkac156_Supplemental_File
