# Supplementary material for: Programmable manipulation of oligonucleotide–albumin interaction for elongated circulation time
Source: Nucleic Acids Res. 2022 Mar 16;50(6):3083–95. doi: 10.1093/nar/gkac156 (PMC8989545; doi:10.1093/nar/gkac156)
Supplement: gkac156_Supplemental_File [file gkac156_supplemental_file.pdf]

SUPPLEMENTARY DATA

Programmable manipulation of oligonucleotide-albumin interaction for elongated circulation time

Cai Yang[1,2,†], Haitao Zhao[1,†], Yang Sun[1,†], Cheng Wang[1], Xinyao Geng[1], Ruowen Wang[1,*], Lumin Tang[1], Da Han[1], Jianjun Liu[1,*], and Weihong Tan [1,2,*]

[1] Institute of Molecular Medicine (IMM), Department of Nuclear Medicine, Institute of Clinical Nuclear Medicine, State Key Laboratory of Oncogenes and Related Genes, Renji Hospital, School of Medicine, Shanghai Jiao Tong University, Shanghai 200127, China; [2] Molecular Science and Biomedicine Laboratory (MBL), State Key Laboratory of Chemo/Biosensing and Chemometrics, College of Chemistry and Chemical Engineering, College of Biology, and Aptamer Engineering Center of Hunan Province, Hunan University, Changsha, Hunan 410082, China.

The synthesis of phosphoramidite F

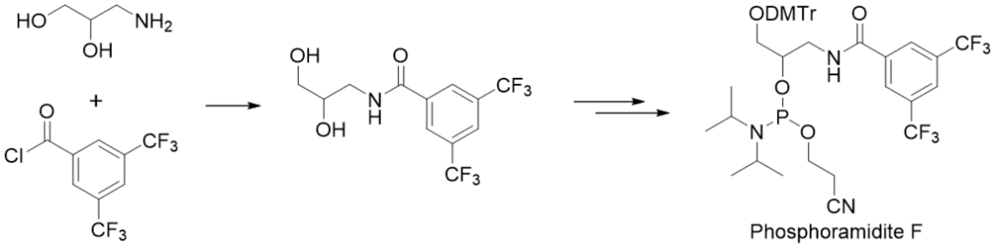

Phosphoramidite F is freshly synthesized and dried under vacuum overnight before the oligo synthesis. From commercially available 3-amino-1,2-propanediol and 3,5- bis(trifluoromethyl) benzoyl chloride following our previous reported protocol[s1], phosphoramidite F is prepared as a white foam. ^{1}H NMR (500 MHz, $CDCl_3$) δ 8.26 (d, *J* = 5.3 Hz, 2H), 8.16 (s, 1H), 7.50 – 7.44 (m, 2H), 7.37 – 7.24 (m, 7H), 7.20 (d, *J* = 7.3 Hz, 1H), 6.85 – 6.79 (m, 4H), 4.26 – 4.12 (m, 1H), 3.75 (d, *J* = 4.2 Hz, 1H), 3.73 (t, *J* = 3.9 Hz, 6H), 3.70 – 3.52 (m, 5H), 3.28 – 2.95 (m, 3H), 2.61 – 2.49 (m, 2H), 1.16 – 1.06 (m, 12H). ^{31}P NMR 149.5, 148.0.

The synthesis of oligonucleotides:

Oligonucleotides were synthesized on an ABI 3900 synthesizer (Applied Biosystems), and then deprotected in AMA (ammonium hydroxide/40% aqueous methylamine, 1:1) at 65 °C for 30 min and further purified by reversed-phase HPLC (Agilent) on a C-18 column (Waters) using 0.1 M triethylamine acetate (TEAA) buffer and acetonitrile as the eluents. The sequences of oligos were listed in Table S1 and the structures were confirmed by mass spectra shown in Supplementary Figure S13-S33.

ssDNA	Sequence (5’-3’)
Sgc8-F10	FATCTAACTGCTGCGCCGCCGGGAAAATACTGTACGGTTAGAF-Fam
Sgc8-F20	FFATCTAACTGCTGCGCCGCCGGGAAAATACTGTACGGTTAGAFF-Fam (Cy5)
Sgc8-F30	FFFATCTAACTGCTGCGCCGCCGGGAAAATACTGTACGGTTAGAFFF-Fam
Sgc8-F40	FFFFATCTAACTGCTGCGCCGCCGGGAAAATACTGTACGGTTAGAFFFF-Fam
Sgc8-F01	ATCTAACTGCTGCGCCGCCGGGAAAFTACTGTACGGTTAGA-Fam
Sgc8-F03	ATCTAACTGCTGCGCCGCCGGGAFAFTAFTGTACGGTTAGA-Fam
Sgc8-F05	ATCTAACTGCTGCGCCGCCGGGFFAFTFFTGTACGGTTAGA-Fam
Sgc8-F07	ATCTAACTGCTGCGCCGCCGGGFFFFFFFTGTACGGTTAGA-Fam

Sgc8-F21	FFATCTAACTGCTGCGCCGCCGGGAAAFTACTGTACGGTTAGAFF-Fam
Sgc8-F23	FFATCTAACTGCTGCGCCGCCGGGAFAFTAFTGTACGGTTAGAFF-Fam (Cy5)
Sgc8-F27	FFATCTAACTGCTGCGCCGCCGGGFFFFFFFTGTACGGTTAGAFF-Fam
Sgc8-D10	(5FU)ATCTAACTGCTGCGCCGCCGGGAAAATACTGTACGGTTAGA-Fam
Sgc8-D01	ATCTAACTGCTGCGCCGCCGGGAAAA(5FU)ACTGTACGGTTAGA-Fam
Sgc8-F23-D10	(5FU)FFATCTAACTGCTGCGCCGCCGGGAFAFTAFTGTACGGTTAGAFF-Fam
Sgc8-F23-D01	FFATCTAACTGCTGCGCCGCCGGGAFAF(5FU)AFTGTACGGTTAGAFF-Fam
Control-F23	FFATACGGTGACTGCGCCGCCGGGAFAFTAFTGTCTAACCGTAFF-Fam
SYL3C	CACTACAGAGGTTGCGTCTGTCCCACGTTGTCATGGGGGGTTGGCCTG-Cy5
SYL3C-F20	FFCACTACAGAGGTTGCGTCTGTCCCACGTTGTCATGGGGGGTTGGCCTGFF-Cy5
P-N-Sgc8-F23	FFATCTAACTGCTGCGCCGCCGGGAFAFTAFTGTACGGTTAGAFF-NH2
P-N-Sgc8	ATCTAACTGCTGCGCCGCCGGGAAAATACTGTACGGTTAGA -NH2
Sgc8-Chol	(Chol)ATCTAACTGCTGCGCCGCCGGGAAAATACTGTACGGTTAGA-Cy5
Sgc8-C18	(C18)TTTTTTATCTAACTGCTGCGCCGCCGGGAAAATACTGTACGGTTAGA-Cy5

Table S1. Detailed sequence information of aptamer derivatives. F represents the artificial base from phosphoramidite F, (5FU) presents fluorouracil, (Chol) represents cholesterol unit by commercial method, (C18) represents a linear lipid chain from the corresponding phosphoramidite[S2].

The synthesis of oligonucleotides conjugated with NOTA and radiolabeling with ^{68}Ga:

Following previously reported protocol[s3], the solution of 1,4,7-triazacyclononane-1,4,7-triacetic acid mono-N-hydroxysuccinimide ester (NHS-NOTA, Macrocyclics, Inc.) in a dry solution of DMSO was added to the buffer of precursor (P-N-Sgc8 and P-N-Sgc8-F23, the oligonucleotides functionalized with NH2) in a sodium phosphate/sodium bicarbonate buffer, and the reaction was stirred at room temperature for 2 hr. Products NOTA-Sgc8 and NOTA-Sgc8-F23 were purified by HPLC, which were radiolabeled with ^{68}Ga by chelation with radioactive $GaCl_3$. The radiolabelling yields were analyzed by HPLC.

Supplementary Figures

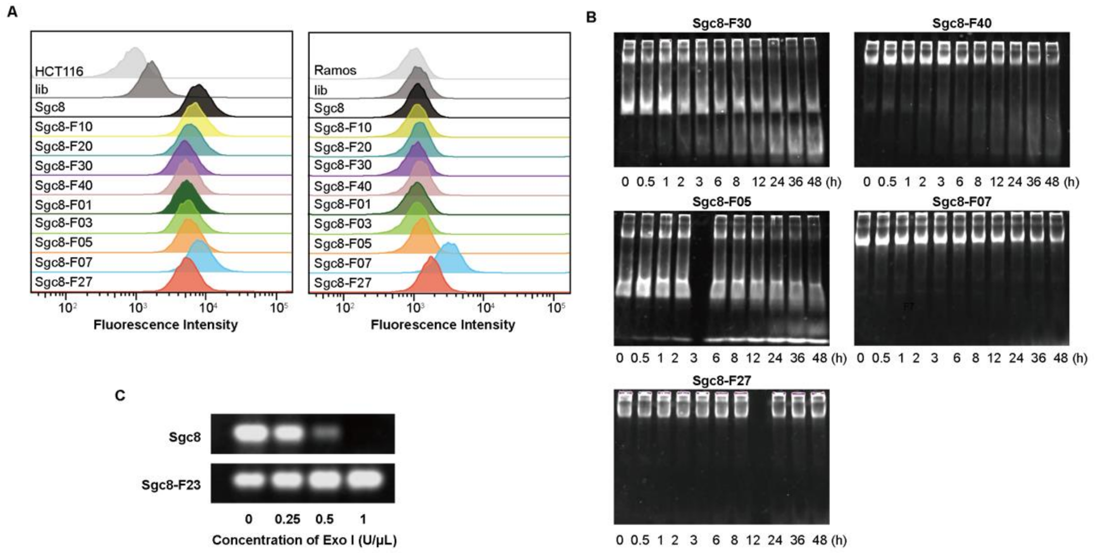

Figure S1. (A) Binding ability to HCT116 cells and Ramos cells tested with flow cytometric assay. Unmodified Sgc8 was used as the positive control, and library (lib) was used as the negative control. The cells were incubated with DNAs respectively at a concentration of 250 nM in a binding buffer for 30min. The incubation temperature was 4 °C. **(B)** Stability analysis of aptamers after incubation with 10% FBS for different times, as determined by 10% PAGE. PAGE experiment demonstrated the non-specific binding with the serum proteins when three or more F bases were added to the terminal of Sgc8 or replaced in the middle. **(C)** Stability analysis of sgc8 (upper) and Sgc8-F23 (lower) after incubation in different concentrations of nuclease (Exo I) in 37℃ for 30 min, as determined by agarose electrophoresis.

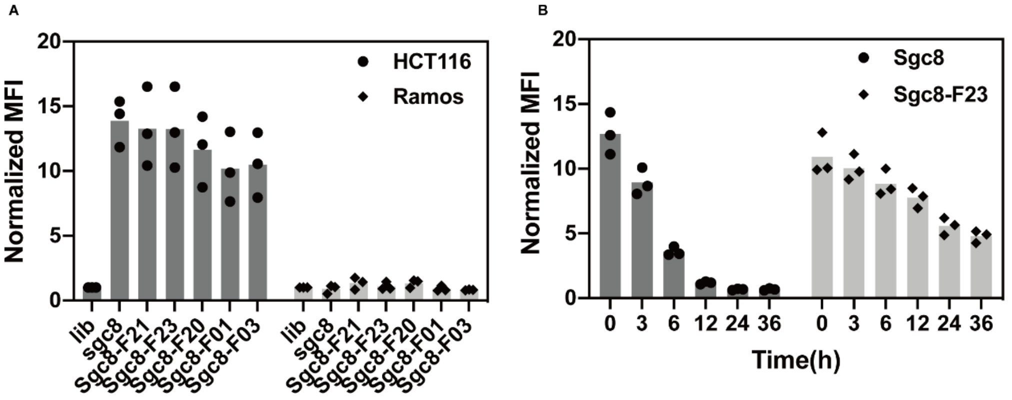

Figure S2. (A) Normalized median fluorescence intensity (MFI) of flow cytometric assay in Figure 2A. **(B)** Normalized median fluorescence intensity (MFI) of flow cytometric assay in Figure 2C.

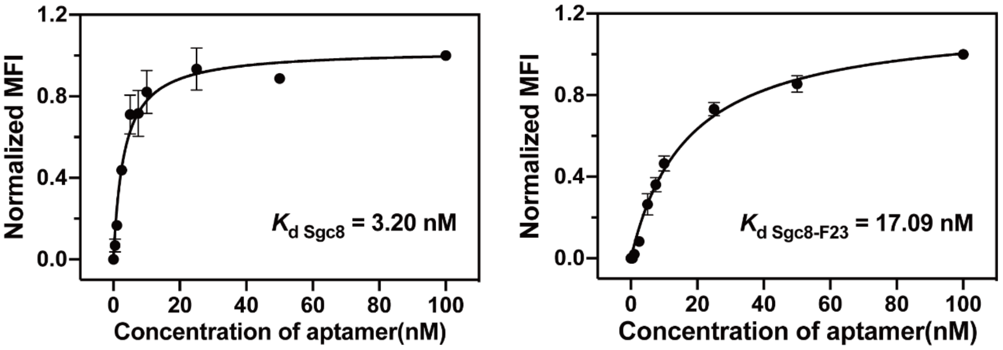

Figure S3. Dissociation constants of Sgc8 and Sgc8-F23 to HCT116 cells. n = 3, error bars denote standard deviation.

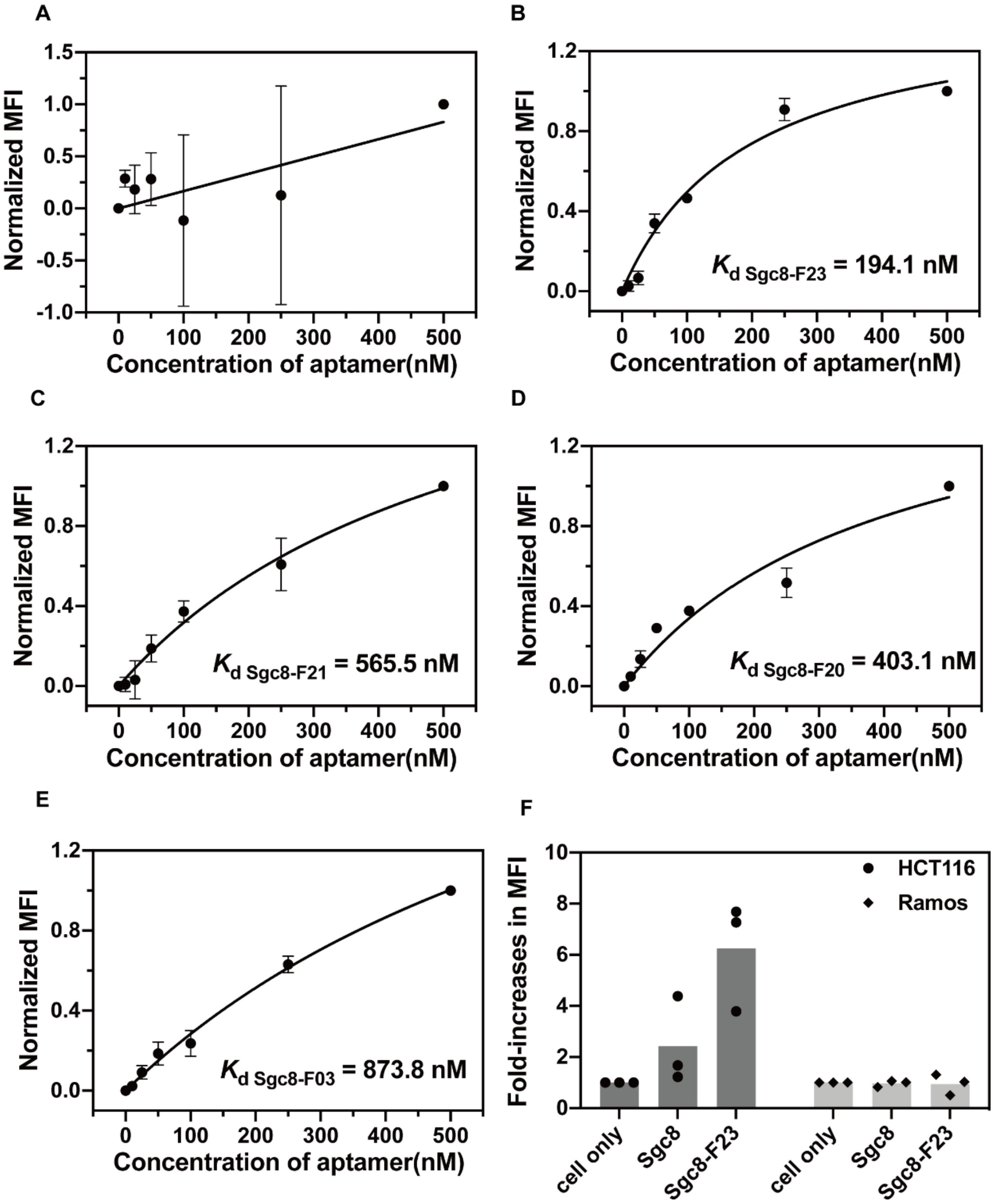

Figure S4. (A-E) Dissociation constants of Sgc8, Sgc8-F23, Sgc8-F21, Sgc8-F20, and Sgc8-

F03 to HSA. n = 3, error bars denote standard deviation. **(F)** Normalized median fluorescence intensity (MFI) of flow cytometric assay in Figure 4B.

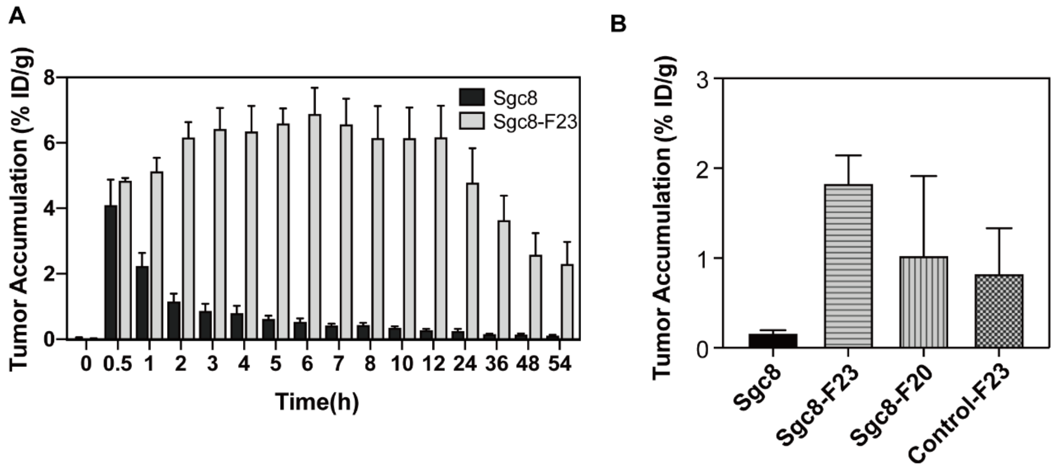

Figure S5. (A) Percentage of Sgc8 or Sgc8-F23 accumulated in the tumor at various time points post-injection, evaluated by measuring the fluorescent intensity of Cy5, and the values were normalized to the injection dosage (ID) and tumor weight. **(B)** Percentage of Sgc8, Sgc8-F23, Sgc8-F20, or Control-F23 accumulated in the tumor at 54 h after injection. n = 3, error bars denote standard deviation.

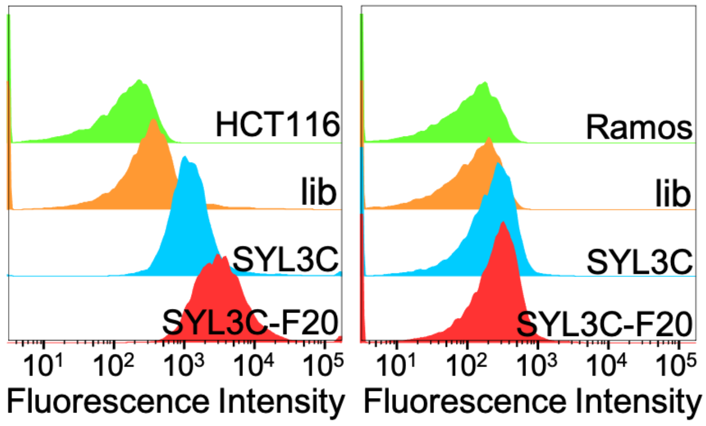

Figure S6. Binding ability to HCT116 cells and Ramos cells tested with flow cytometric assay. Unmodified SYL3C was used as a positive control, and library (lib) was used as a negative control. The cells were incubated with DNAs respectively at a concentration of 250 nM in a binding buffer for 30min. The incubation temperature was 4 °C.

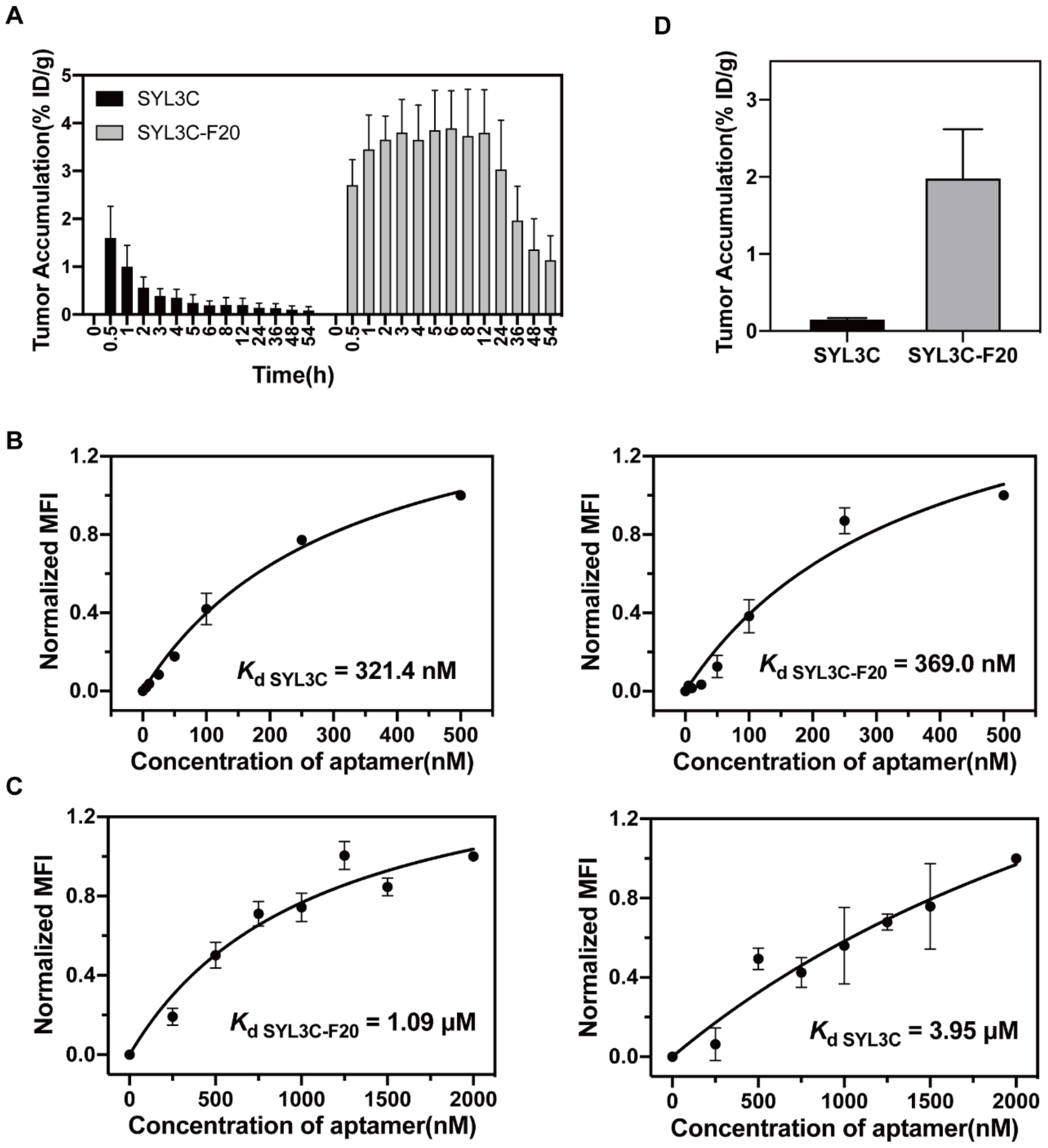

Figure S7. (A) Percentage of SYL3C or SYL3C-F20 accumulated in the tumor at various time points post-injection, evaluated by measuring the fluorescent intensity of Cy5, and the values were normalized to the injection dosage (ID) and tumor weight. **(B)** Dissociation constants of SYL3C and SYL3C-F20 to HCT116 cells. **(C)** Dissociation constants of SYL3C and SYL3C-F20 to HSA. **(D)** Percentage of SYL3C and SYL3C-F20 accumulated in the tumor at 54 h after injection. n = 3, error bars denote standard deviation.

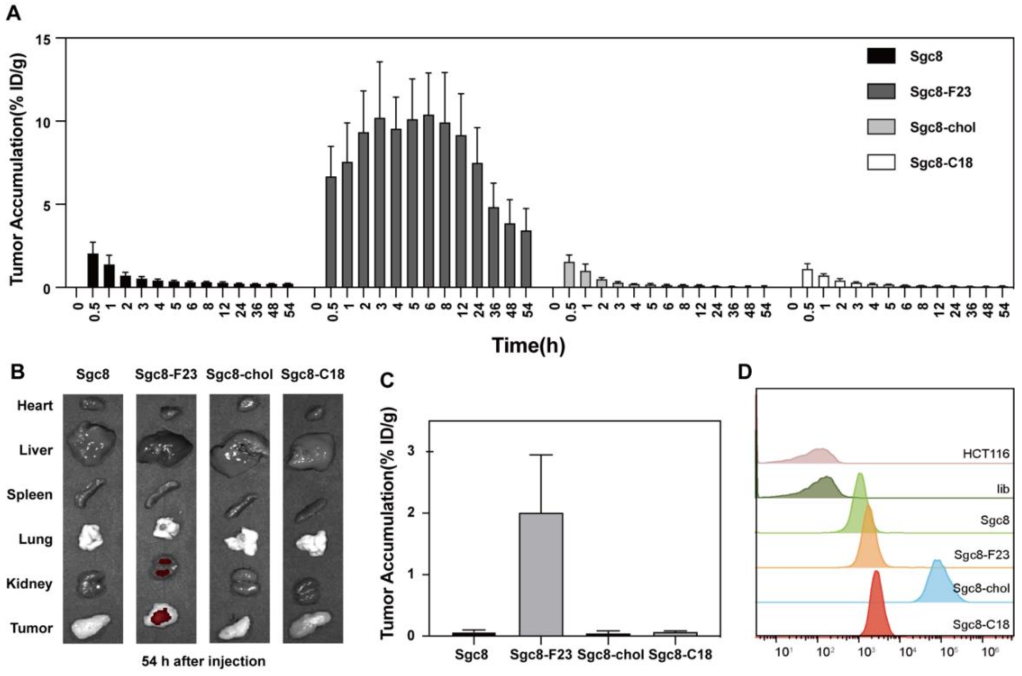

Figure S8. (A) Percentage of Sgc8, Sgc8-F23, Sgc8-chol, and Sgc8-C18 accumulated in the tumor at various time points post-injection, evaluated by measuring the fluorescent intensity of Cy5, and the values were normalized to the injection dosage (ID) and tumor weight. **(B)** The distribution of the Sgc8, Sgc8-F23, Sgc8-chol, and Sgc8-C18 in tumor and major organs (heart, liver, spleen, lung, kidney) at 54 h after injection visualized by fluorescence imaging. **(C)** Percentage of SYL3C and SYL3C-F20 accumulated in the tumor at 54 h after injection. n = 3, error bars denote standard deviation. **(D)** The binding ability of Cy5-labeled Sgc8-F23, Sgc8-chol, and Sgc8-C18 to HCT116 cells was tested with flow cytometric assay. Unmodified Sgc8 was used as a positive control, and library (lib) was used as a negative control. The cells were incubated with DNAs respectively at a concentration of 250 nM in a binding buffer for 30min. The incubation temperature was 4 °C.

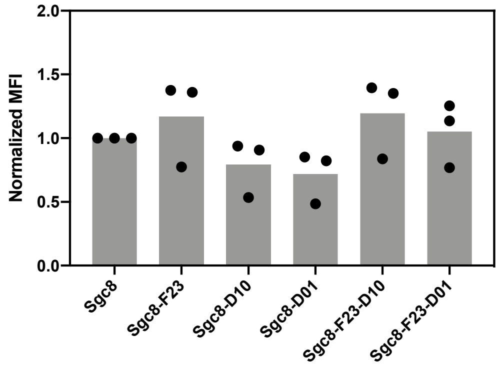

Figure S9. Normalized median fluorescence intensity (MFI) of flow cytometric assay in Figure 9A.

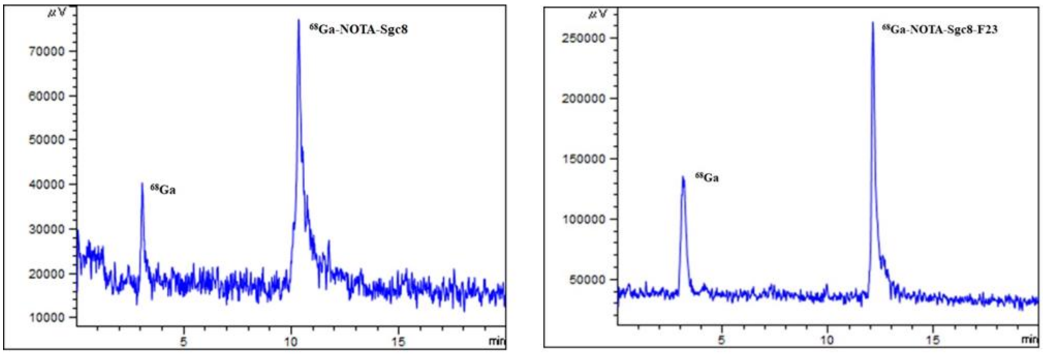

Figure S10. HPLC spectra of ^{68}Ga-NOTA-Sgc8 and ^{68}Ga-NOTA-Sgc8-F23, radiolabelling yields of which are 85% and 70% respectively.

Copies of NMR Spectra:

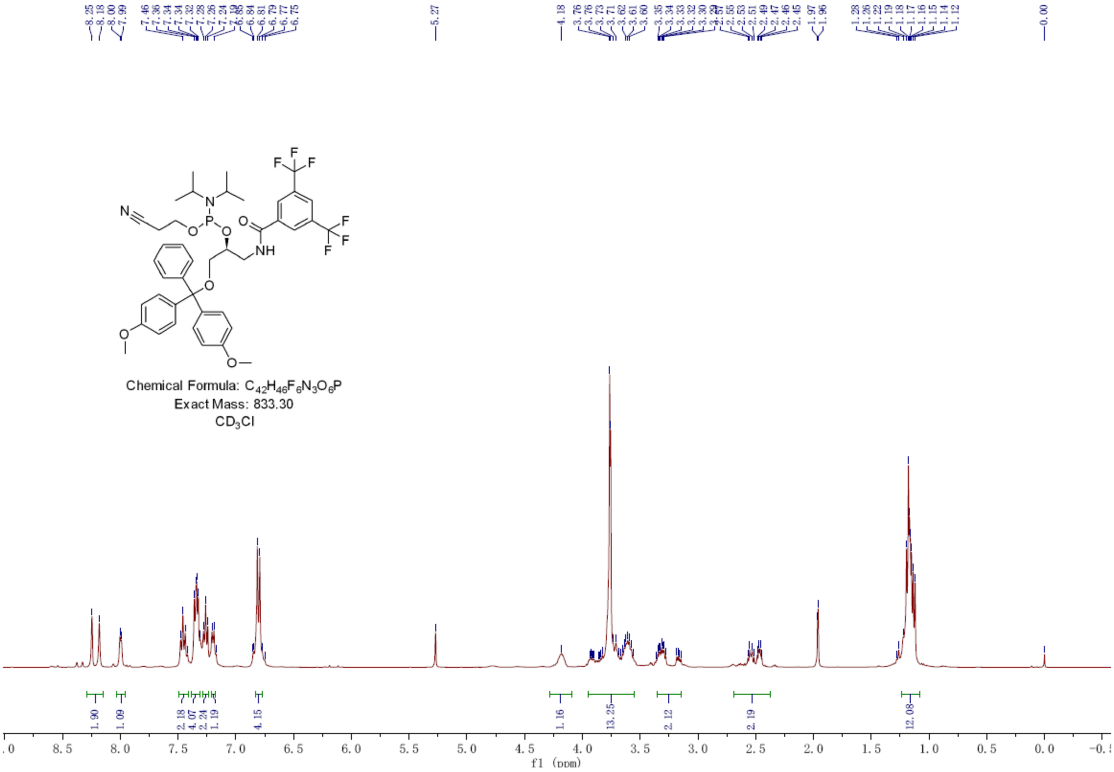

Figure S11. ^{1}H NMR spectrum of phosphoramidite **F**.

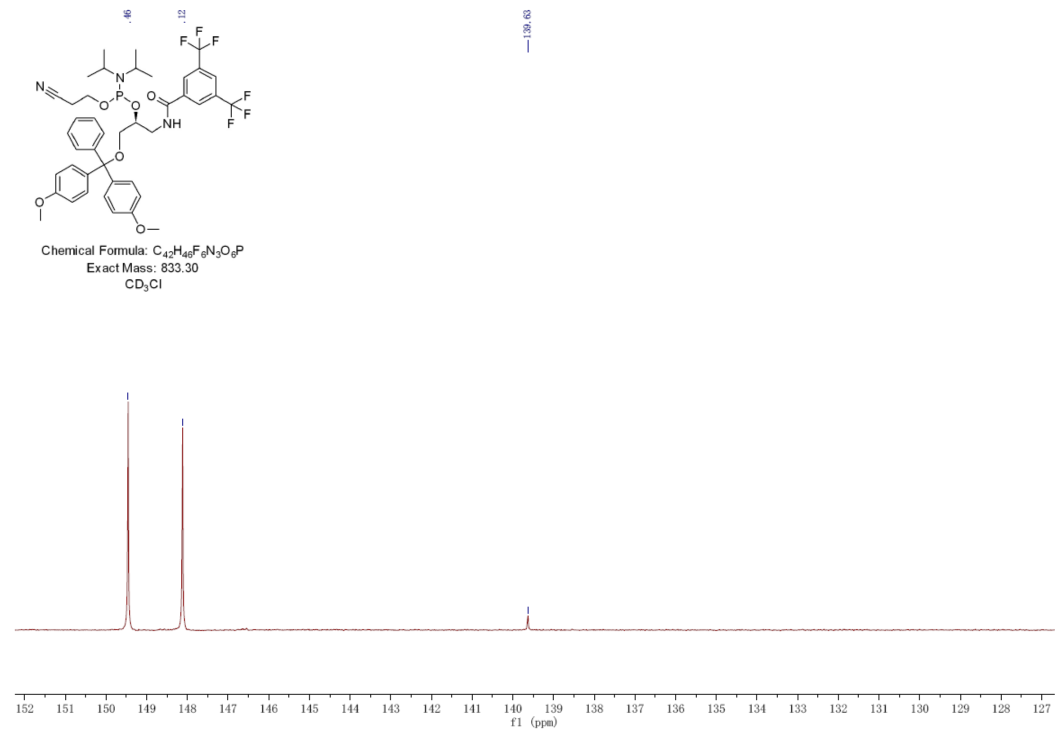

Figure S12. ^{31}P NMR spectrum of phosphoramidite **F**.

Copies of Mass Spectra:

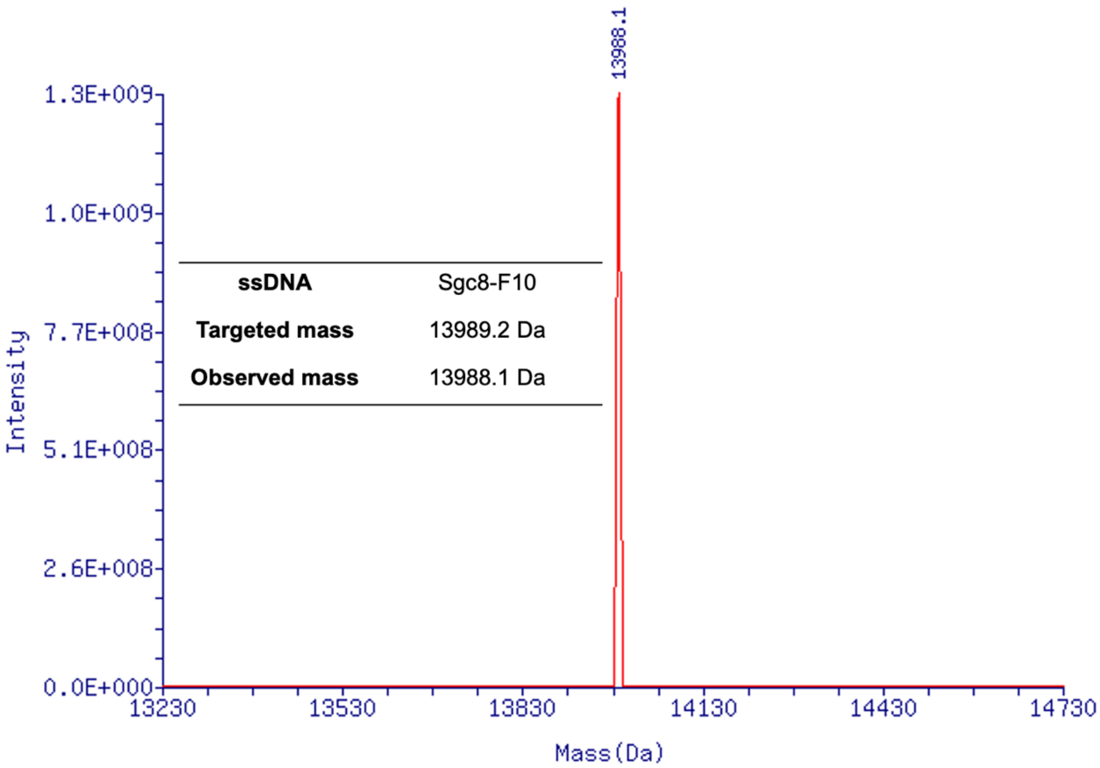

Figure S13. ESI-MS analysis of Sgc8-F10 by Sangon (Shanghai). Calculated molecular weight was 13989.2, and observed DNA peak was 13988.1.

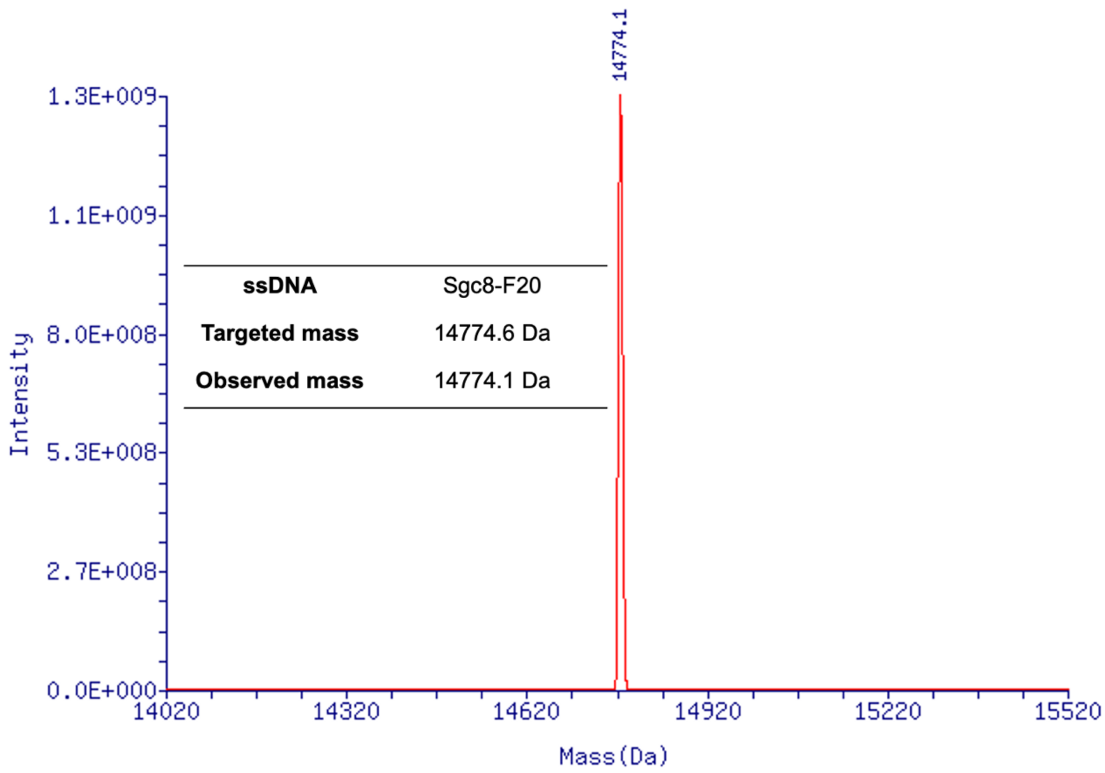

Figure S14. ESI-MS analysis of Sgc8-F20 by Sangon (Shanghai). The calculated molecular weight was 14774.6, and the observed DNA peak was 14774.1.

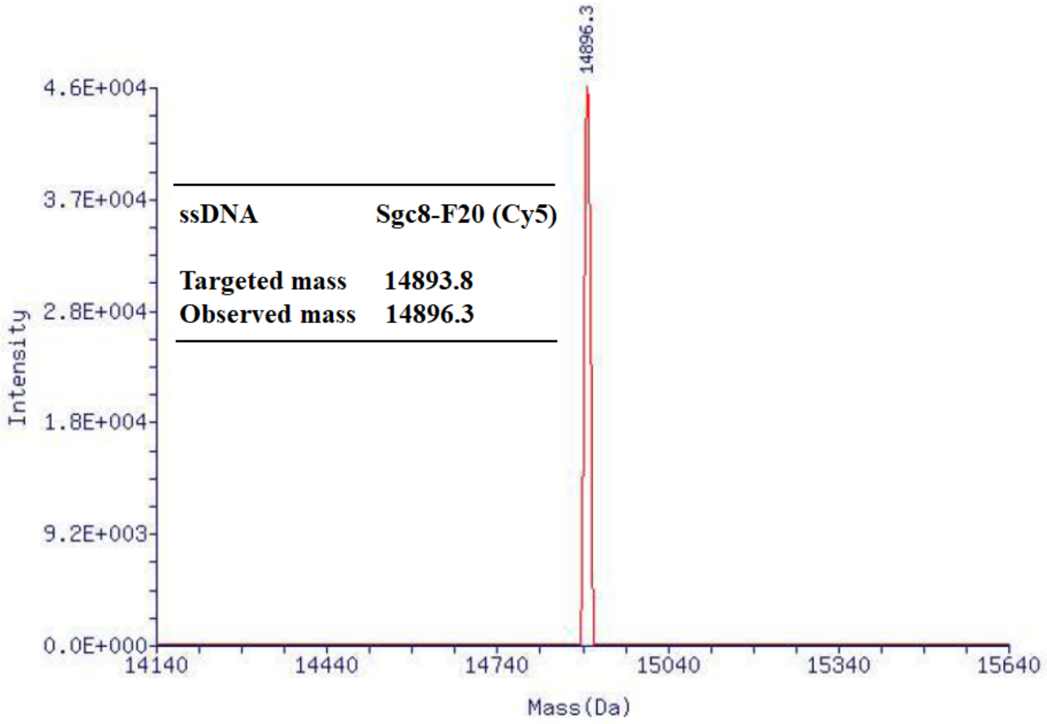

Figure S15. ESI-MS analysis of Sgc8-F20 labeled with Cy5 by Sangon (Shanghai). The calculated molecular weight was 14893.8, and the observed DNA peak was 14896.3.

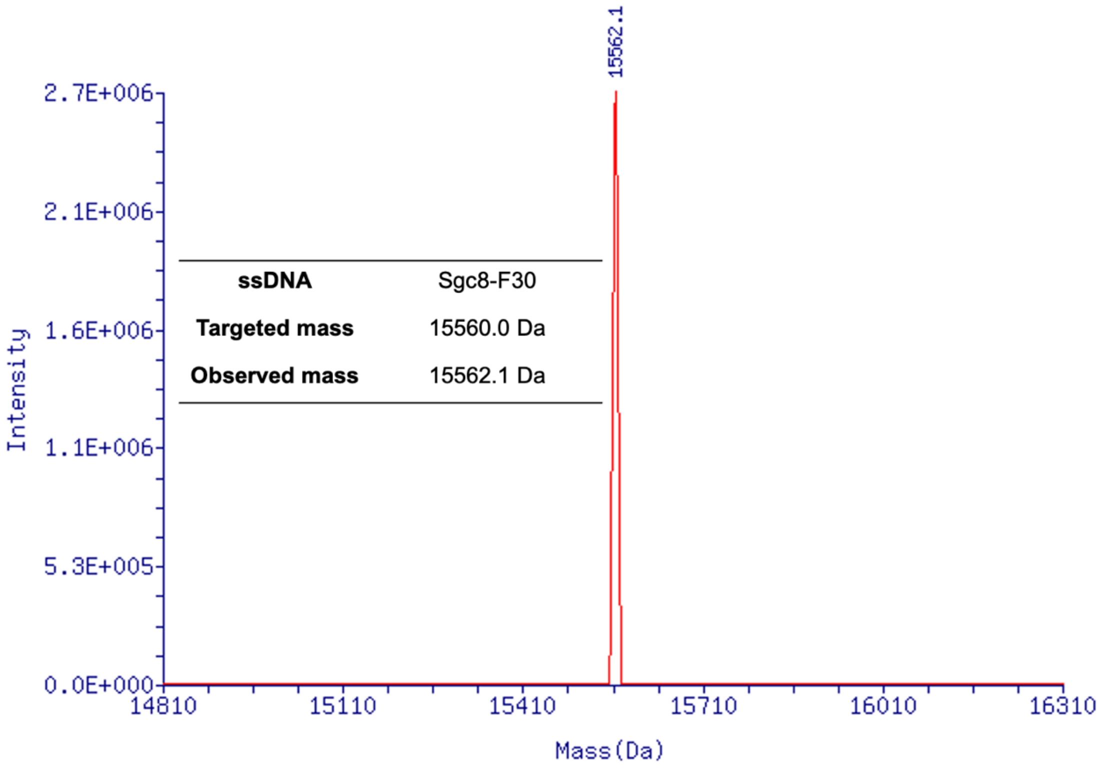

Figure S16. ESI-MS analysis of Sgc8-F30 by Sangon (Shanghai). The calculated molecular weight was 15560.0, and the observed DNA peak was 15562.1.

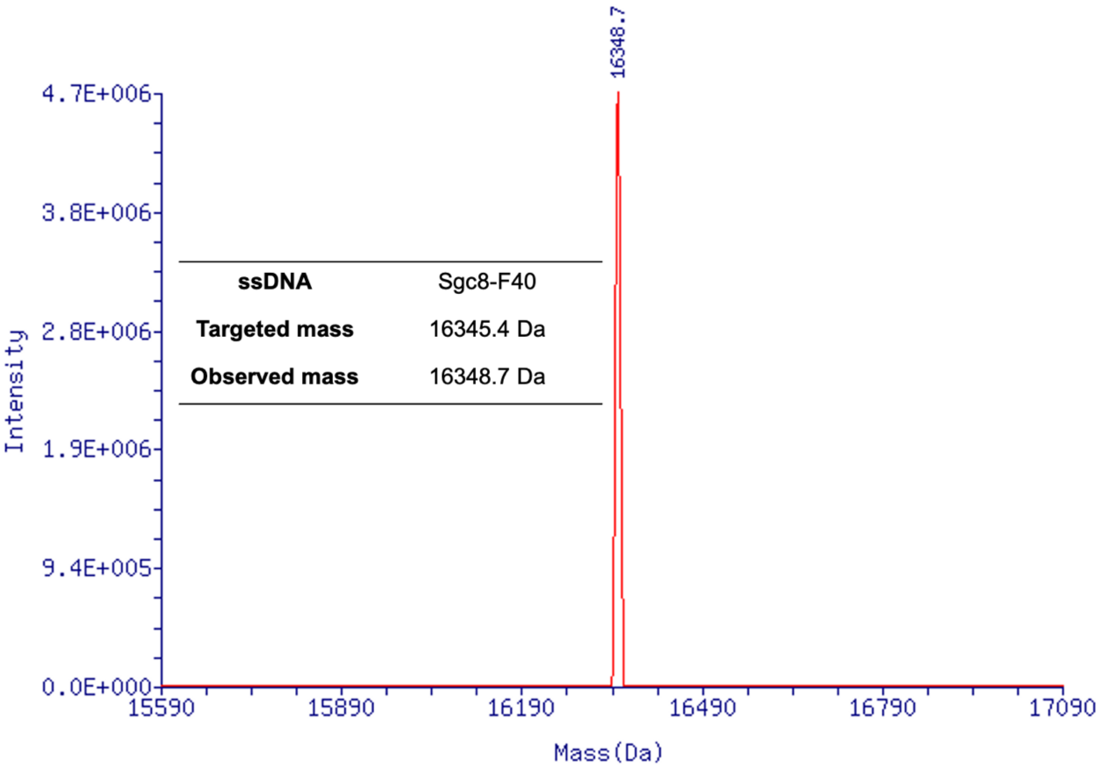

Figure S17. ESI-MS analysis of Sgc8-F40 by Sangon (Shanghai). The calculated molecular weight was 16345.4, and the observed DNA peak was 16348.7.

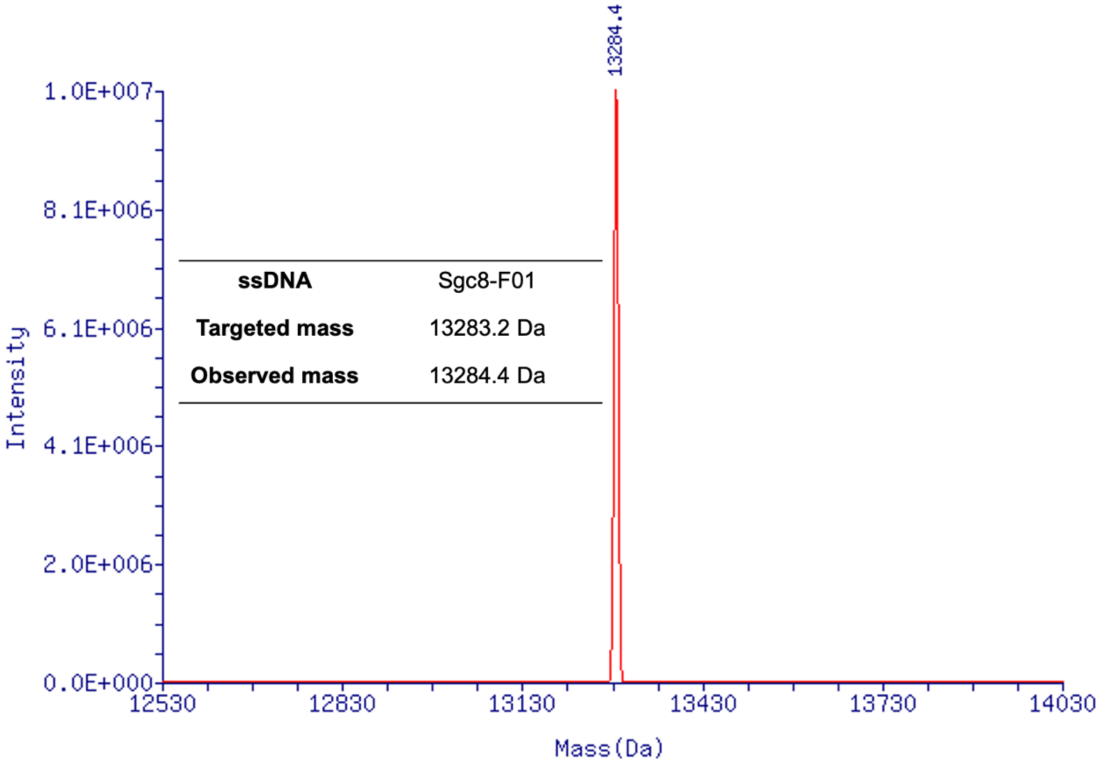

Figure S18. ESI-MS analysis of Sgc8-F01 by Sangon (Shanghai). The calculated molecular weight was 13283.2, and the observed DNA peak was 13284.4.

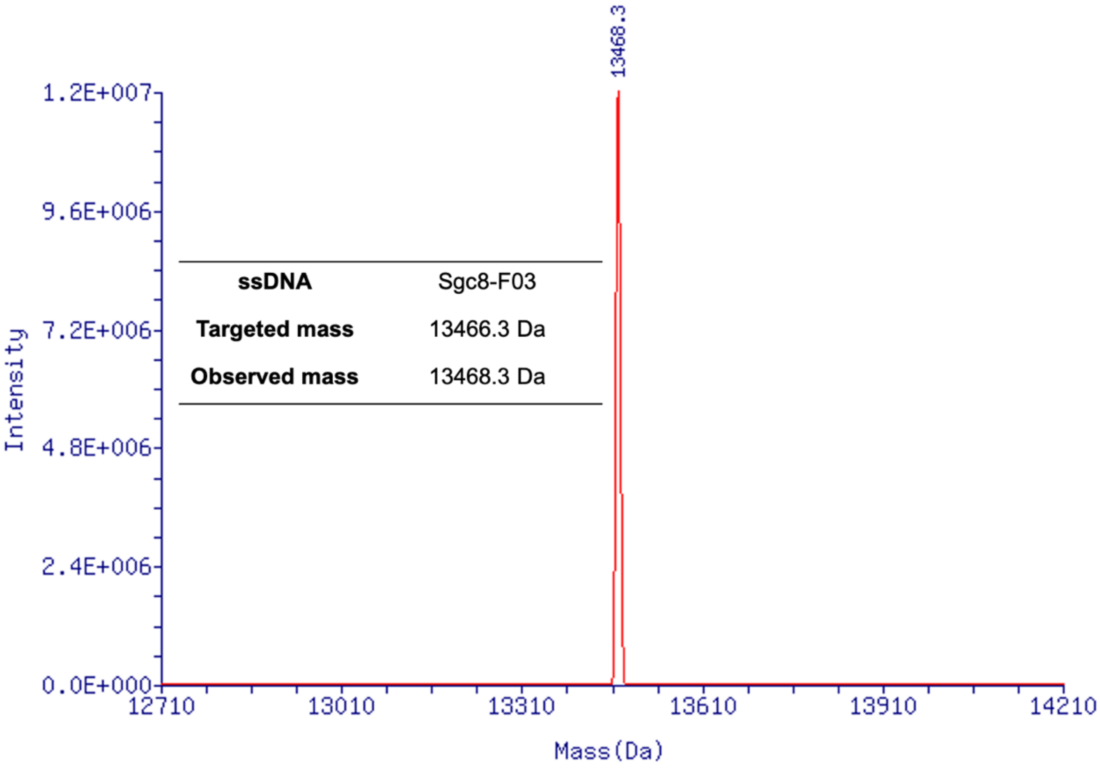

Figure S19. ESI-MS analysis of Sgc8-F03 by Sangon (Shanghai). The calculated molecular weight was 13466.3, and the observed DNA peak was 13468.3.

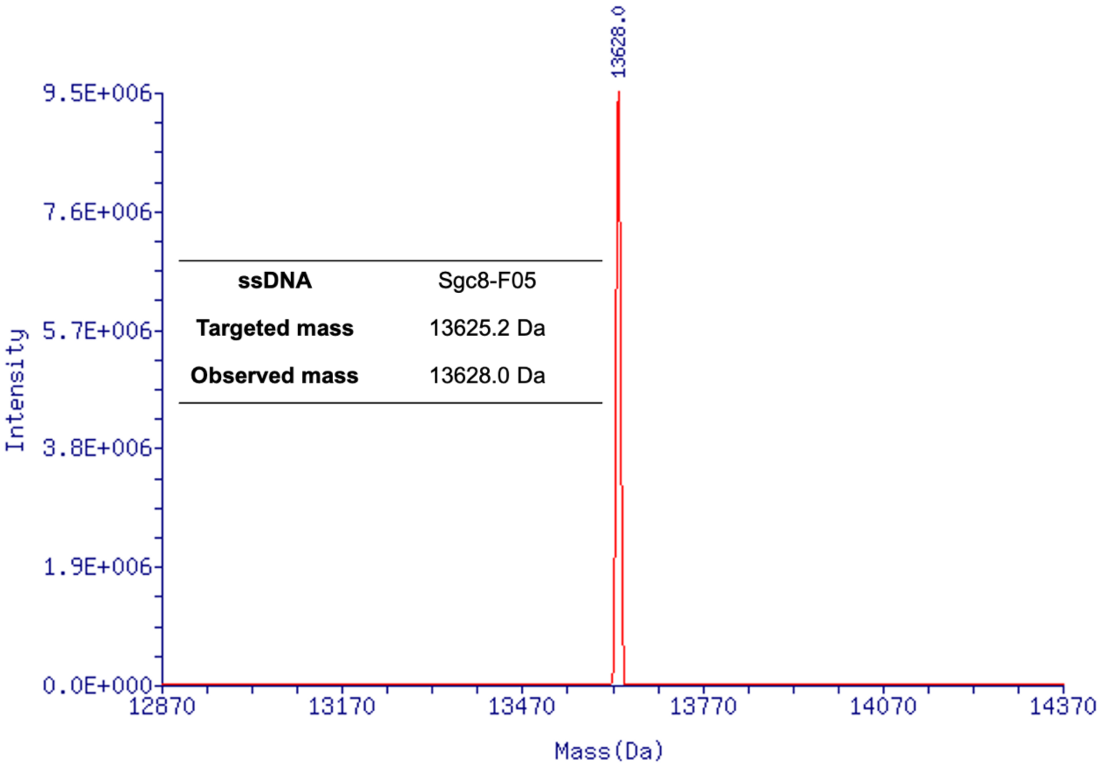

Figure S20. ESI-MS analysis of Sgc8-F05 by Sangon (Shanghai). The calculated molecular weight was 13625.2, and the observed DNA peak was 13628.0.

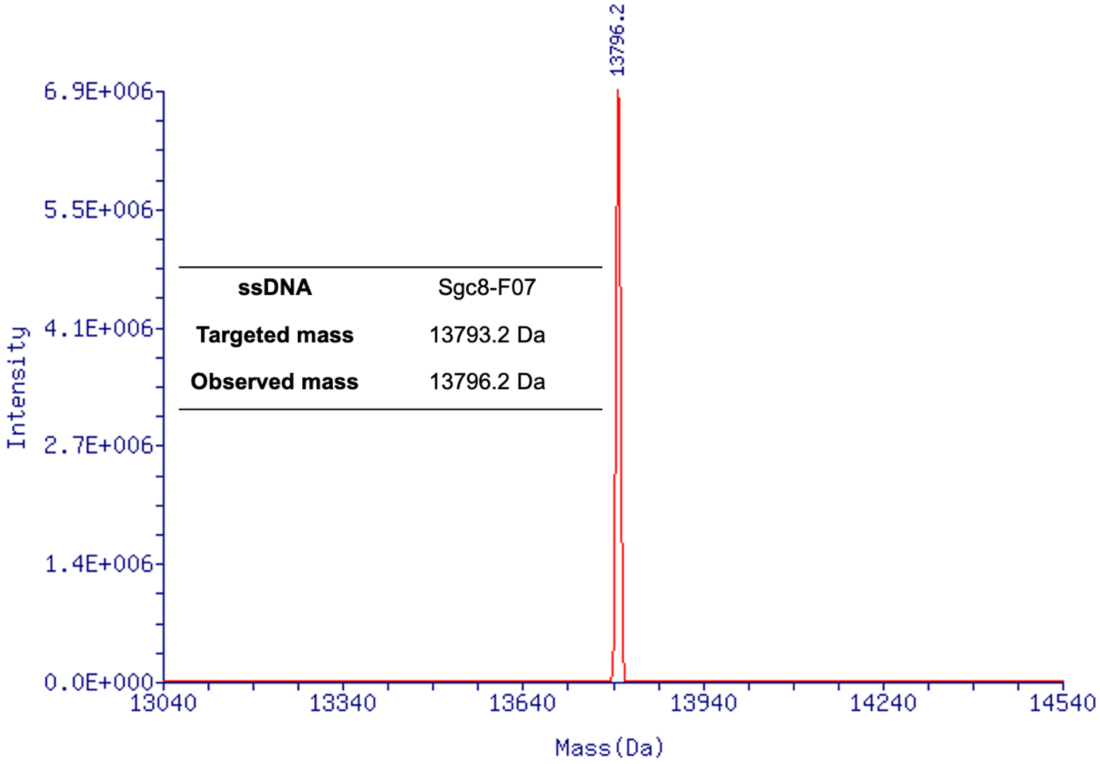

Figure S21. ESI-MS analysis of Sgc8-F07 by Sangon (Shanghai). The calculated molecular weight was 13793.2, and the observed DNA peak was 13796.2.

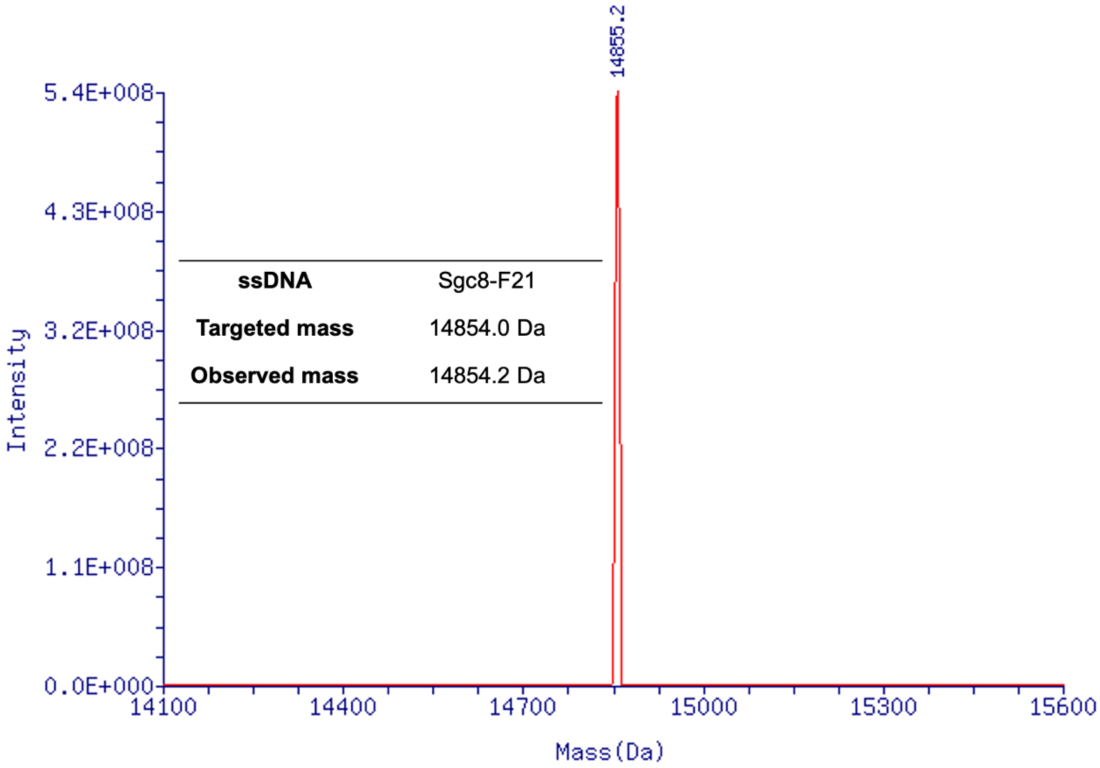

Figure S22. ESI-MS analysis of Sgc8-F21 by Sangon (Shanghai). The calculated molecular weight was 14854.0, and the observed DNA peak was 14854.2.

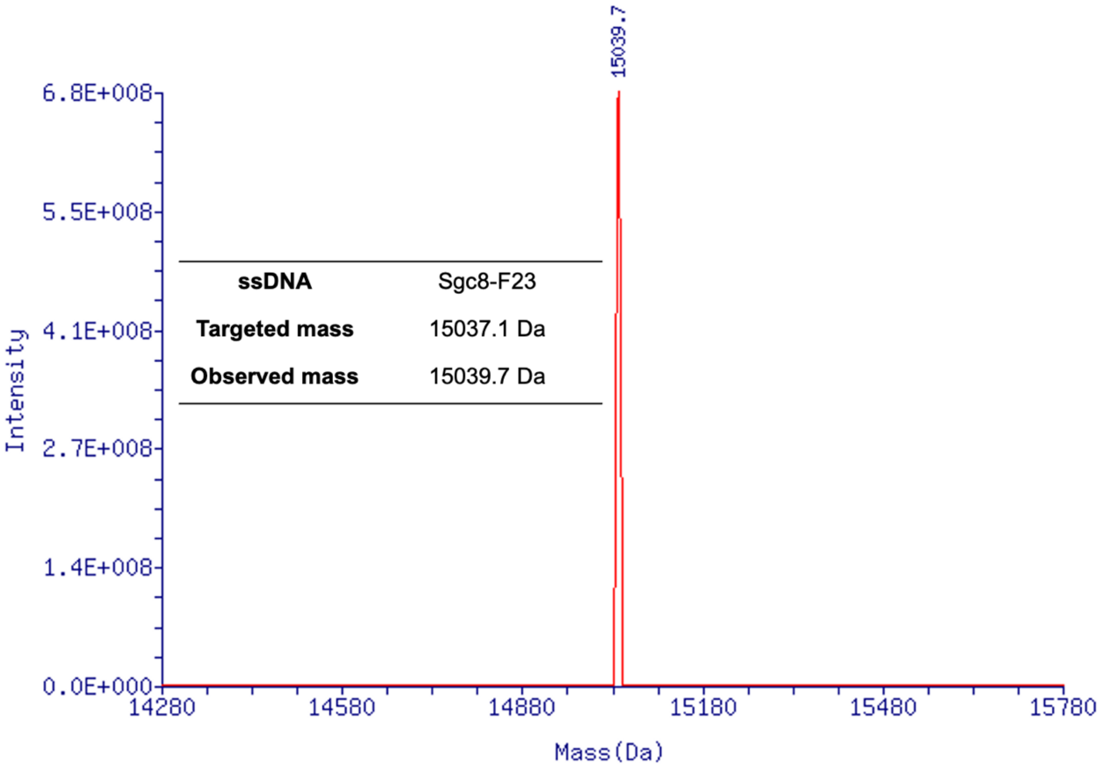

Figure S23. ESI-MS analysis of Sgc8-F23 by Sangon (Shanghai). The calculated molecular weight was 15037.1, and the observed DNA peak was 15039.7.

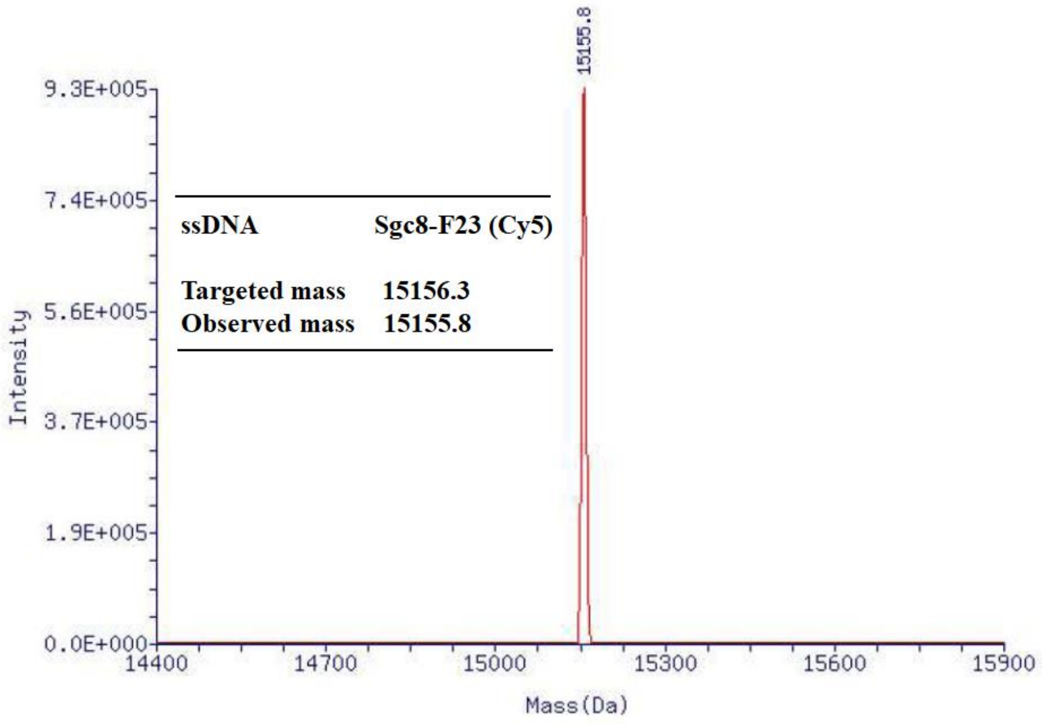

Figure S24. ESI-MS analysis of Sgc8-F23 labeled with Cy5 by Sangon (Shanghai). The calculated molecular weight was 15156.3, and the observed DNA peak was 15155.8.

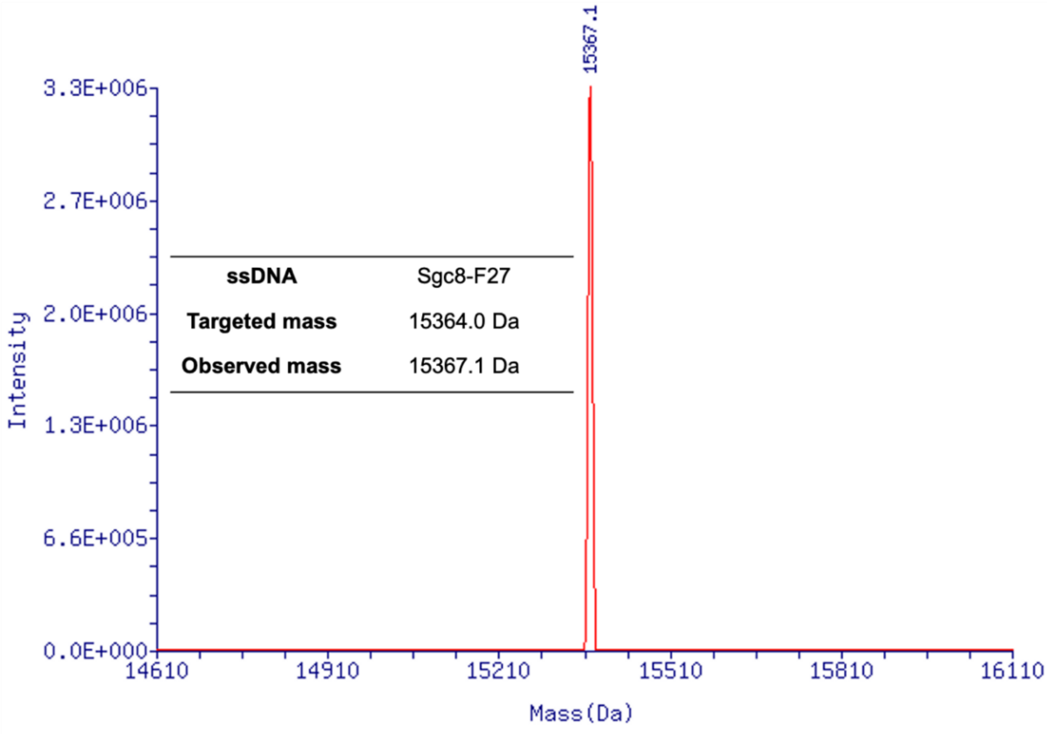

Figure S25. ESI-MS analysis of Sgc8-F27 by Sangon (Shanghai). The calculated molecular weight was 15364.0, and the observed DNA peak was 15367.1.

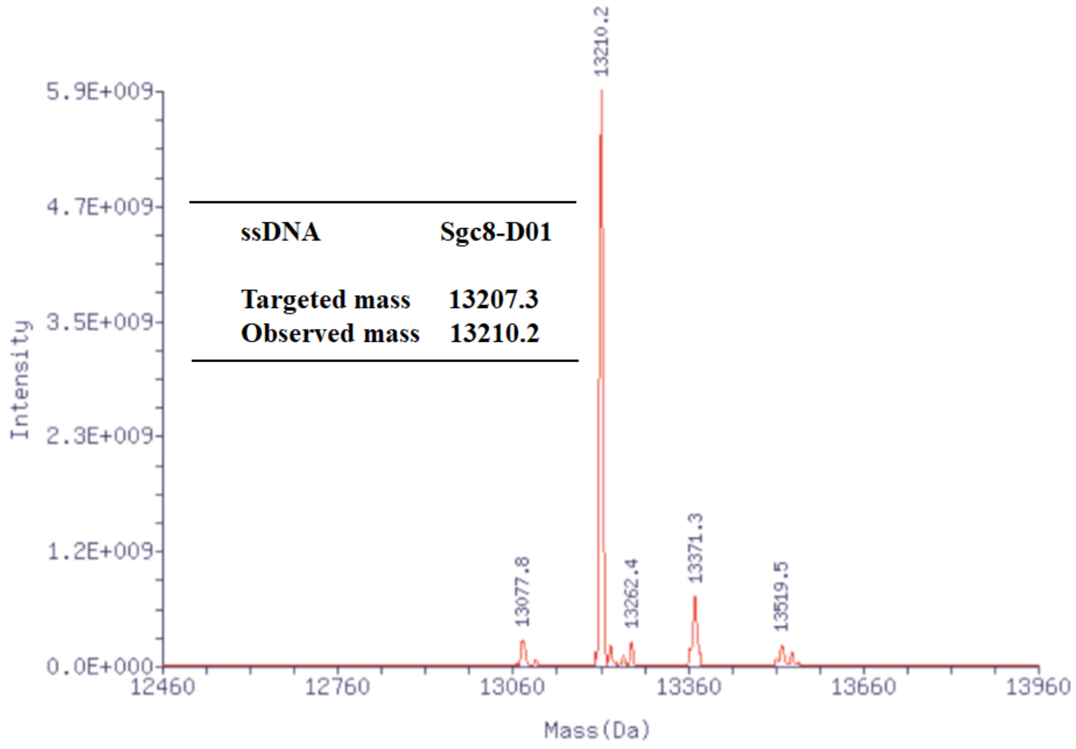

Figure 26. ESI-MS analysis of Sgc8-D01 by Suzhou Biosyntech. The calculated molecular weight was 15364.0, and the observed DNA peak was 15367.1

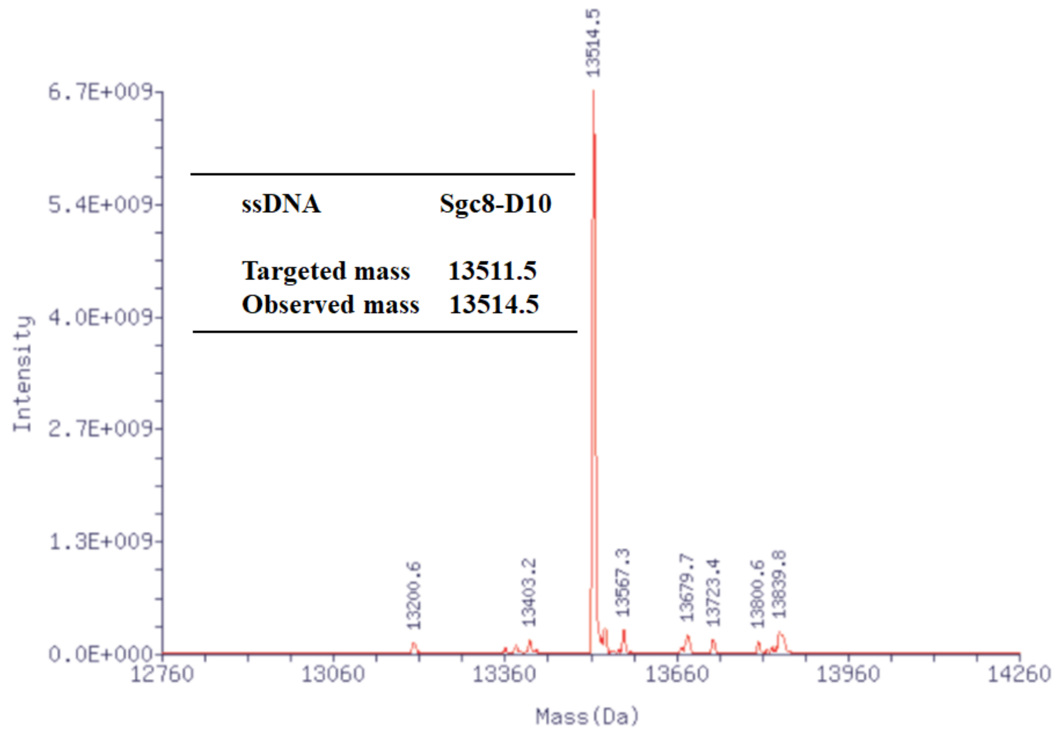

Figure 27. ESI-MS analysis of Sgc8-D10 by Suzhou Biosyntech. The calculated molecular weight was 15311.5, and the observed DNA peak was 13514.5

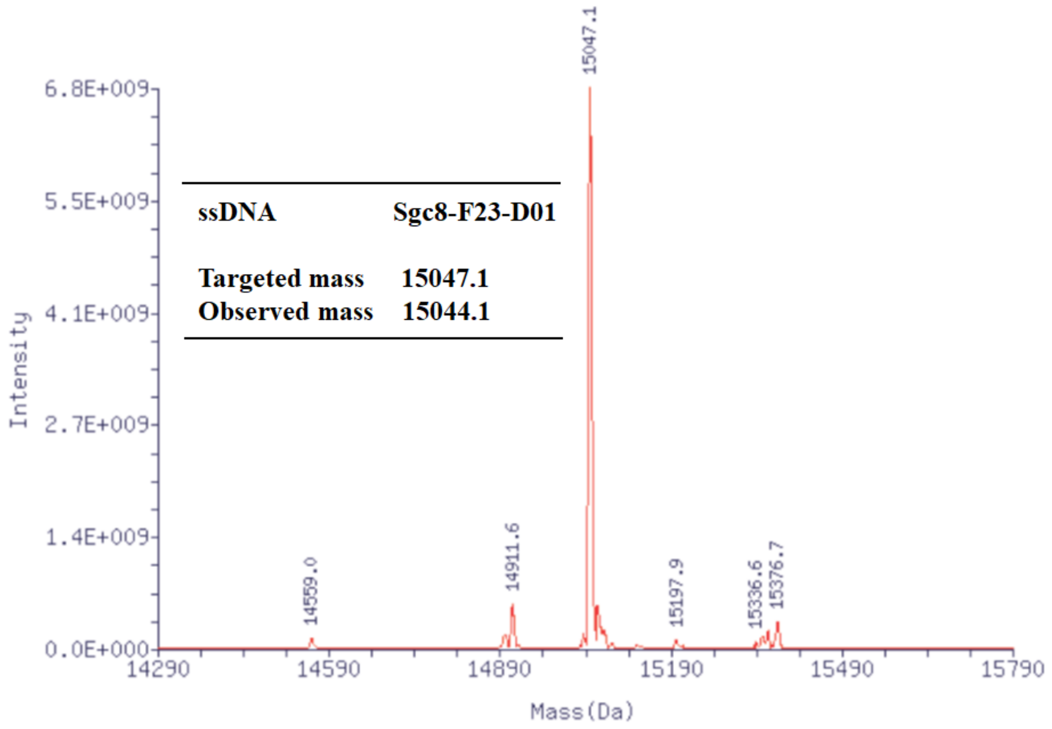

Figure 28. ESI-MS analysis of Sgc8-F23-D01 by Suzhou Biosyntech. The calculated molecular weight was 15047.1, and the observed DNA peak was 15044.1

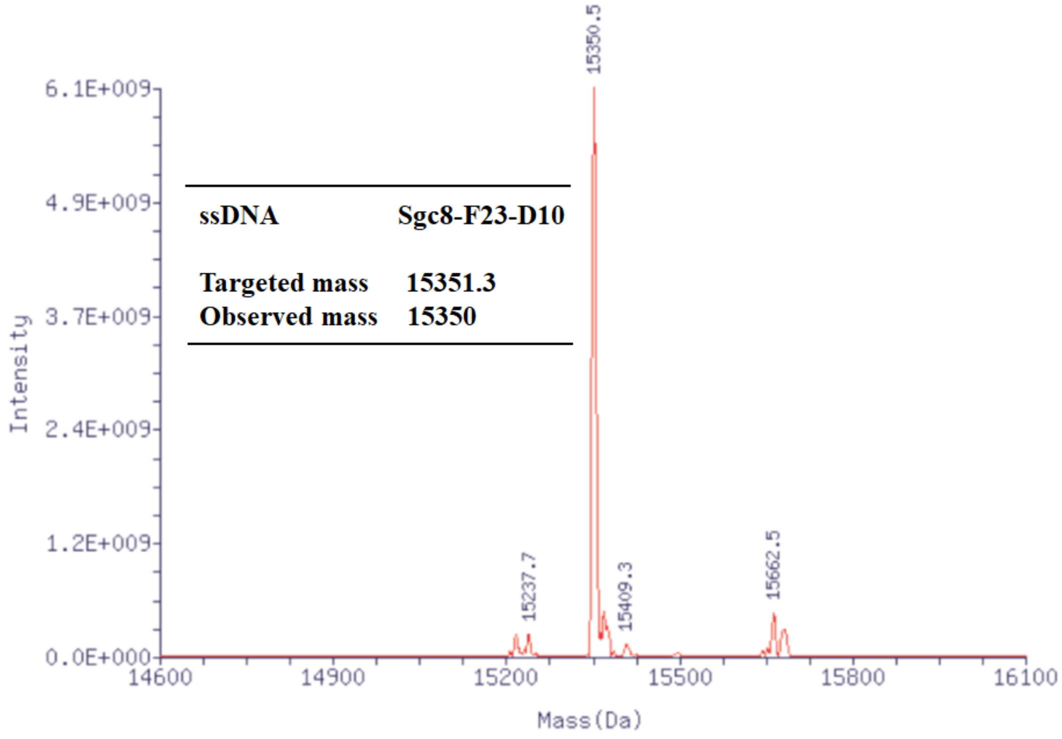

Figure 29. ESI-MS analysis of Sgc8-F23-D10 by Suzhou Biosyntech. The calculated molecular weight was 15351.3, and the observed DNA peak was 15350.5

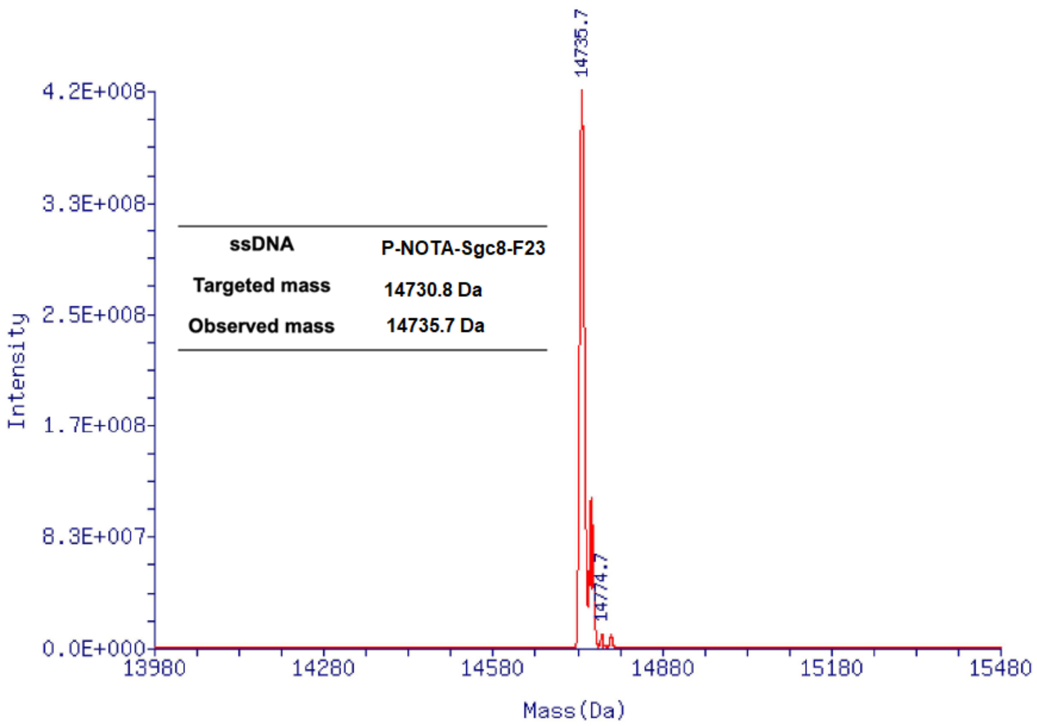

Figure S30. ESI-MS analysis of P-NOTA-Sgc8-F23 by Sangon (Shanghai). The calculated molecular weight was 14730.8, and the observed DNA peak was 14735.7.

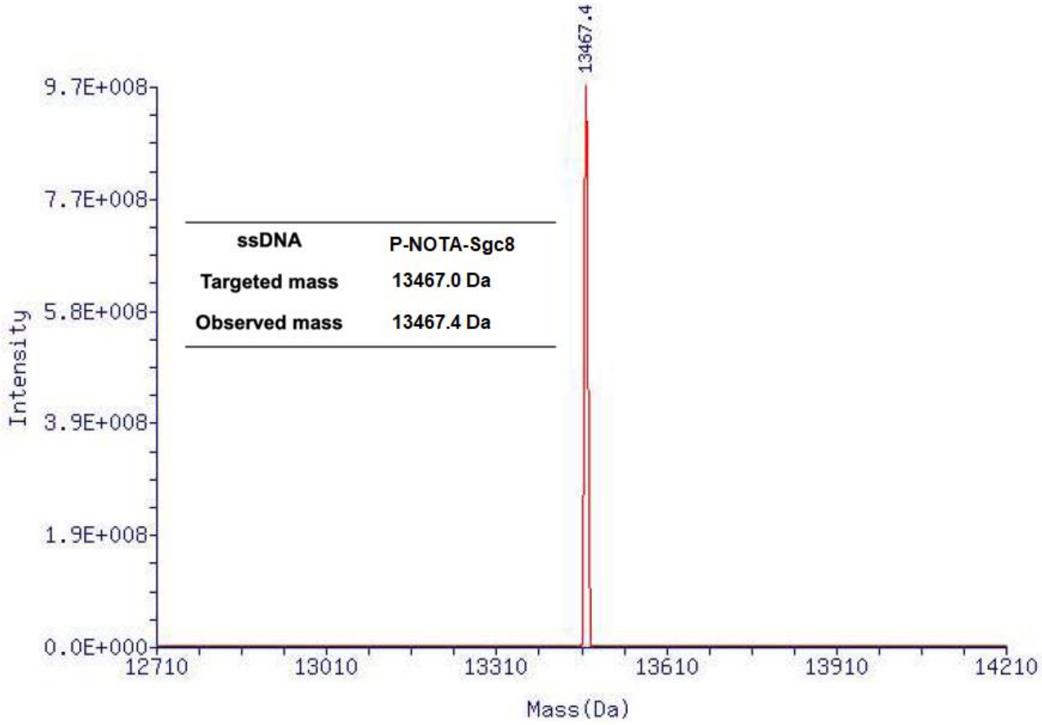

Figure S31. ESI-MS analysis of P-NOTA-Sgc8 by Sangon (Shanghai). The calculated molecular weight was 13467.0, and the observed DNA peak was 13467.4.

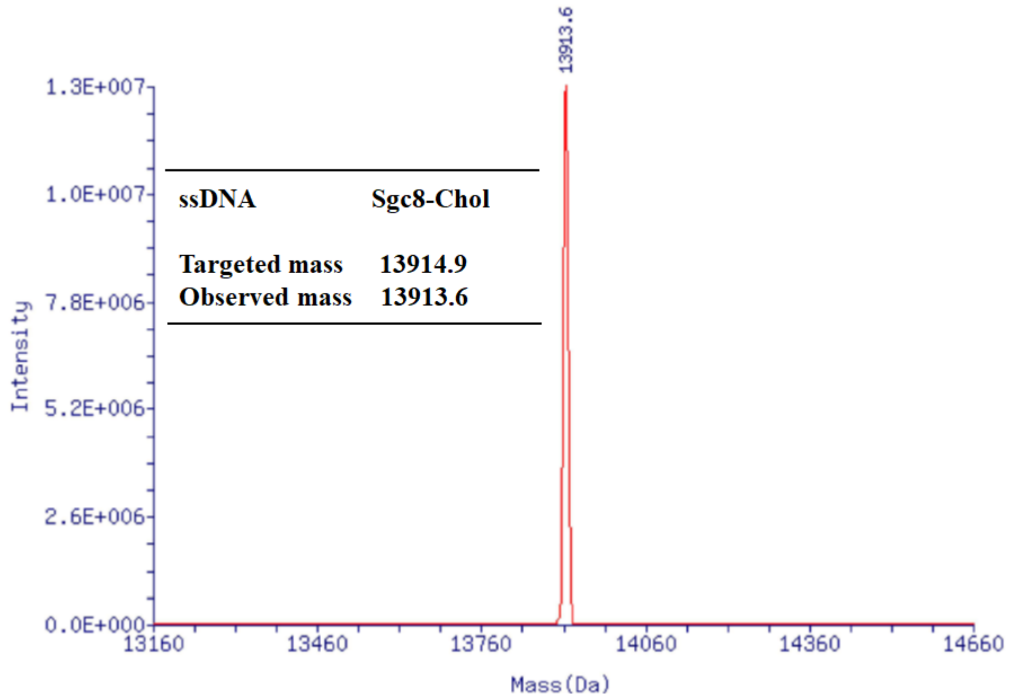

Figure S32. ESI-MS analysis of Sgc8-Chol by Sangon (Shanghai). The calculated molecular weight was 13914.9, and the observed DNA peak was 13913.6.

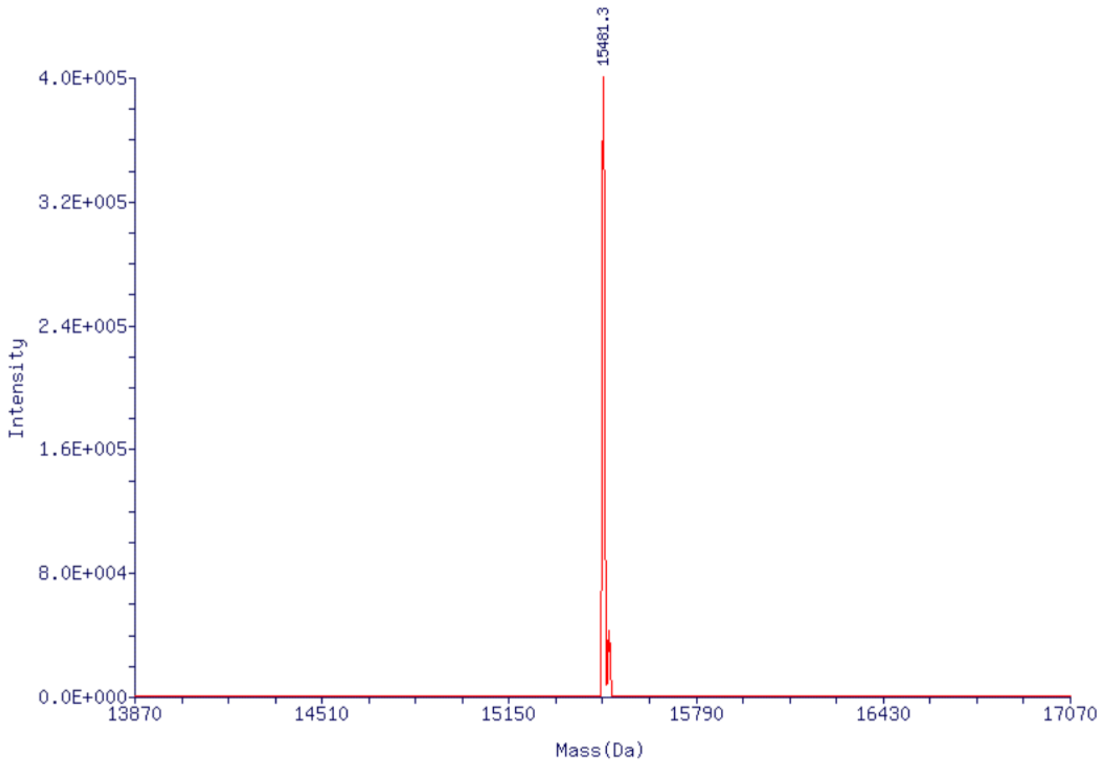

Figure 33. ESI-MS analysis of Sgc8-C18 by Suzhou Biosyntech. The calculated molecular weight was 15479.9, and the observed DNA peak was 15481.3

Reference:

S1. Wang, R., Wang, C., Cao, Y., Zhu, Z., Yang, C., Chen, J., Qing, F.L. and Tan, W. Trifluoromethylated Nucleic Acid Analogues Capable of Self-Assembly through Hydrophobic Interactions. *Chem Sci*, **2014** *5*, 4076.

S2. Tian, L., Shao, M., Gong, Y., Chao, Y., Wei, T., Yang, K., Chen, Q., Liu, Z. Albumin-binding lipid-aptamer conjugates for cancer immunoimaging and immunotherapy. *Sci. China Chem.* 2021, Online. https://doi.org/10.1007/s11426-021-1168-4.

S3. Sicco, E., Baez, J., Margenat, J., Garcia, F., Ibarra, M., Cabral, P., Moreno, M., Cerecetto, H., Calzada, V. Derivativations of Sgc8-c aptamer to prepare metallic radiopharmaceuticals as imaging diagnostic agents: Syntheses, isolations, and physicochemical characterizations. *Chem. Biol. Drug. Des.* **2018**, *91, 747.*
